# Mitophagy’s impacts on cancer and neurodegenerative diseases: implications for future therapies

**DOI:** 10.1186/s13045-025-01727-w

**Published:** 2025-08-01

**Authors:** Jason Huang, Vincent Truong Pham, Shaozi Fu, Gang Huang, Ya-Guang Liu, Lei Zheng

**Affiliations:** 1 Department of Pathology and Laboratory Medicine, The University of Texas Health Science Center at San Antonio, San Antonio, TX 78229 USA; 2 MD Anderson Mays Cancer Centerat , UT Health San Antonio, San Antonio, TX 78229 USA; 3 Department of Cell Systems and Anatomy, UT Health San Antonio, San Antonio, TX 78229 USA; 4 Department of Thoracic Surgery, The First Affiliated Hospital of Zhejiang University School of Medicine, Hangzhou, 310003 Zhejiang China

**Keywords:** Mitophagy, Mitochondrial dysfunction, Cancer, Neurodegenerative diseases, Inflammation, ROS, Mutations, Cell death, Calcium, Therapeutics

## Abstract

Substantial evidence supports an inverse relationship between cancer and neurodegenerative diseases (NDDs), but few studies investigate the biological mechanisms underlying this phenomenon**.** While previous explanations—such as inflammation, reactive oxygen species (ROS), genetic mutations, and cell death—remain significant, they ultimately converge on mitophagy. This review identifies mitophagy as a pivotal factor in the development of both cancer and NDDs, while also evaluating specific mechanisms and processes to clarify how mitophagy connects these opposing disease trajectories. By examining these factors, we aim to uncover the underlying mechanisms that explain the inverse relationship between cancer and NDDs, which will help develop therapeutic strategies that target common factors for both conditions.

**Supplementary Information:**

The online version contains supplementary material available at 10.1186/s13045-025-01727-w.

## Introduction

The average human lifespan has experienced a remarkable surge, increasing from 30 to 80 years in less than a century [1]. However, this increase in longevity has also brought a rise in aging-related diseases. Cardiovascular disease (CVD), neurodegenerative diseases (NDDs), and cancer are now the three most prominent aging-related conditions [2]. In the latter two conditions, a distinct pattern has emerged: individuals often develop either NDDs or cancer, but rarely both. In fact, individuals who develop one are far less likely to develop the other [3–7]. This intriguing inverse correlation has drawn scientific interest and warrants further investigation. Understanding the biological mechanisms behind this relationship could reveal key insights into the onset and progression of both diseases, and potentially lead to novel, targeted therapies.

Tumorigenesis is a highly dynamic and complex process driven by genetic, epigenetic, metabolic, and microenvironmental factors [8, 9]. Cancer develops through the accumulation of genetic mutations and epigenetic modifications alongside complex interactions between other intrinsic and extrinsic factors. These changes disrupt critical biological pathways that regulate cell proliferation, apoptosis, DNA repair, immune surveillance, and cellular metabolism [10–16]. Furthermore, cancer comprises a heterogeneous population of cells characterized by diverse malignant hallmarks, plasticity, and evolutionary adaptability, which collectively contribute to disease progression, metastasis, and therapeutic resistance [8, 9, 11, 17–21]. Similarly, NDDs are complex disorders characterized by progressive neuronal dysfunction and degeneration, ultimately leading to cognitive, motor, and behavioral impairments. The pathogenesis of both these diseases involves interplay between genetic mutations, RNA dysregulation, pathological protein aggregation, mitochondrial dysfunction, oxidative stress, neuroinflammation, glial activation, and synaptic and neuronal network dysfunction [22–26]. However, unlike cancer, which is defined by uncontrolled cellular proliferation, NDDs primarily target specific neuronal populations and their niches, leading to irreversible neuronal loss and functional decline. These distinctions highlight fundamental similarities and differences in cell fate determination and disease progression between cancer and NDDs [22, 25].

The inverse correlation between cancer and NDDs likely reflects intrinsic links to cell fate determinants and the distinct pathophysiological mechanisms that govern these disease states and hallmarks. Mitochondria are indispensable for maintaining cellular homeostasis and mammalian life. Due to their endosymbiotic origin and diverse functions in energy production, metabolism, apoptosis, calcium homeostasis, and cellular signaling (the mitochondrial information processing system, MIPS), mitochondria are positioned at the core of cell survival and adaptation, ultimately influencing human health and disease [27–29]. Notably, mitochondria represent the only human genetic system that integrates key hallmarks of age-related diseases such as cancer and NDDs [27, 30]. Therefore, exploring mitochondrial dysfunction in these conditions may provide critical insights into the mechanisms underlying their inverse correlation.

It is important to note that while mitochondrial dynamics, such as fission and fusion, play critical roles in maintaining mitochondrial function, these topics have been comprehensively reviewed elsewhere in the context of cancer and NDDs [31–36]. In this review, we focus specifically on mitophagy—a downstream quality control mechanism that directly reflects and responds to broader mitochondrial stress. Among mitochondrial processes, mitophagy is uniquely situated at the intersection of cell survival and cell death [37–39], making it a compelling lens through which to examine the inverse correlation between cancer and NDDs. We explore the shared and opposing molecular mechanisms between tumorigenesis and NDD pathogenesis, emphasizing the role of mitochondrial dysfunction and mitophagy as key factors in this inverse correlation. We do this by exploring a context provided by epidemiological studies, then dissecting the specific biological processes and mechanisms within mitophagy, as well as how they relate to both tumorigenesis and NDD pathogenesis. Through this, we highlight how mitochondrial dysfunction and mitophagy function as converging pathways in both diseases, before finally discussing the therapeutic implications of mitophagy-based interventions, exploring their potential as novel strategies for treating both cancer and NDDs.

### Epidemiological studies between cancer and NDDs

The unexpected relationship between cancer and NDDs—two conditions that appear to be biological opposites—challenges traditional views of disease pathology. This relationship underscores the complexity of their underlying mechanisms and highlights the intricate network of cellular processes involved in their initiation and progression [40]. Understanding the existing evidence driving this connection is crucial for unraveling the mechanisms at play. Therefore, it is essential to acknowledge the role of epidemiological studies before exploring potential biological mechanisms. These studies provide valuable, tangible evidence for the inverse relationship between cancer and NDDs and provide a foundation of data to make biological hypotheses from. Considering the extensive range of NDDs, this discussion will primarily focus on Alzheimer's Disease (AD) and Parkinson's Disease (PD), two well-known aging-related conditions that collectively account for approximately 16% of all NDD cases in the United States. Focusing on well-studied examples of NDDs allows us to consider the broader implications of our conclusions for NDDs as a whole.

AD is the most widespread NDD, with a total of 6.2 million diagnoses in the US alone and 24 million diagnoses worldwide. AD is characterized by neurofibrillary tangles and brain plaques formed by phosphorylated tau (p-Tau) protein deposits [41]. Most studies on the relationship between AD and cancer indicate an inverse one, with multiple studies indicating that individuals exhibit a remarkable decline in their susceptibility to cancer after they’ve been diagnosed with AD, with risk reduction estimates ranging from 42 to 50%. Likewise, individuals diagnosed with cancer display a lower likelihood of developing AD, experiencing a decrease in risk ranging from 35 to 37% [42–44]. There is also existing data for specific cancers that showed notable correlations with reduced risk for AD: lung cancer (9% reduction), leukemia (2.4% reduction), and breast cancer (5.9% reduction) [45]. An intriguing association between AD and cigarette smoking also warrants attention: despite being a well-established risk factor for lung cancer, cigarette smoking appears to exert a neuroprotective effect. The neuroprotective effect appears to result from nicotine’s ability to promote neuron survival, reduce neuronal damage, and decrease neuronal excitotoxicity [46], a sharp contrast with the 15–30 × increase in risk for lung cancer.

Notably, the reduced cancer risk observed in patients with AD includes both smoking-related and non-smoking-related cancers [47]. This challenges a recent hypothesis suggesting that lower smoking rates among AD patients might explain the decreased cancer incidence, given the inverse relationship between smoking and AD risk. While this could potentially account for fewer smoking-related cancers, it fails to explain the reduction in non-smoking-related cancers. This inconsistency highlights the complexity of the inverse association between cancer and NDDs and emphasizes the need for further research to uncover the underlying biological mechanisms.

PD is the second most prevalent NDD, characterized by a staggering one million diagnoses in the United States and ten million diagnoses worldwide [48, 49], and features its unique epidemiological data. PD manifests largely as a progressive movement disorder characterized by the presence of cytoplasmic inclusions comprised of insoluble protein aggregates and a loss of dopaminergic neurons in the substantia nigra [50]. Numerous case–control and cohort studies have consistently reported a reduced risk of nearly all types of cancer among individuals with PD [51]. A decade ago, the prevailing evidence did not indicate an inverse relationship between cancer and NDDs [52]. In fact, they were frequently considered as opposite conditions, with cancer defined by uncontrollable cellular proliferation and NDDs by uncontrolled cellular apoptosis [52–54]. However, the epidemiological studies explored have put forth a contrasting perspective, shedding light on their intriguing connection. This connection can also be extended beyond the realm of epidemiology and into the intricate biological and genetic makeup of both NDDs and tumors. It may also hold translational value for therapeutic strategy. If a shared biological mechanism influences whether a cell progresses toward proliferation or degeneration, then further exploration into this process could offer therapeutic strategies adaptable to both cancer and NDDs, as suggested by epidemiological evidence. In the subsequent sections, the compiled evidence and literature regarding various mechanisms and their potential roles in explaining the inverse relationship between cancer and NDDs will be reviewed. The aim is to collectively analyze these findings and compare them to past hypotheses that can contribute to a deeper understanding of the biological relationship between tumorigenesis and NDDs. Through this comprehensive review, a clearer understanding may emerge, paving the way for a more nuanced explanation of the inverse relationship and informing potential therapies for both conditions.

### The biological mechanisms underlying the inverse correlation

#### Mitochondria: a common link between cancer and NDDs

The biological mechanisms underlying the inverse correlation between cancer and NDDs remain complex and incompletely understood, but the epidemiological evidence serves as a foundation that necessitates further exploration. Specifically, exploring several key biological similarities and differences between the diseases may help explain this phenomenon. From the perspective of cell fate and trajectory, dysregulation of the cell cycle leading to uncontrolled cell division is a hallmark of cancer, often accompanied by defects in apoptosis and cellular senescence [11, 55]. In contrast, NDDs are characterized by increased apoptosis and impaired cell cycle re-entry, contributing to progressive neurodegeneration [22, 25]. At the molecular level, cancer development is frequently driven by defects in the DNA damage response (DDR) and repair pathways, resulting in genomic instability and tumorigenesis. A spectrum of multi-omic alterations—including genomic, epigenomic, epitranscriptomic, and metabolomic changes, as well as dysregulated signaling pathways—contribute to malignant transformation, immune evasion and tumor progression [11, 55]. Conversely, in NDDs, molecular defects drive pathological protein aggregation, age-related cellular dysfunction (e.g., cognitive and motor decline), progressive neuronal loss, and chronic neuroinflammation mediated by microglial activation [22, 25]. These fundamental differences in cell fate determination and pathophysiology may underlie the opposing nature of cancer and NDDs. Notably, when analyzing their shared pathophysiological mechanisms, mitochondrial dysfunction emerges as a common hallmark of both cancer and NDDs, playing a fundamental role in shaping their disease trajectories [11, 22, 25, 55–57].

Mitochondria are unique organelles central to bioenergetics, biosynthetic metabolism, and MIPS. They play a pivotal role in shaping the metabolomic landscape and serve as a key determinant for both physiological functions and pathological conditions [29, 55, 56, 58–61]. Carcinogenesis is a multistep process involving the transformation of normal cells into neoplastic tumor cells. Although the mechanisms driving tumor initiation, promotion, and progression are varied, complex, and sometimes unclear, metabolic reprogramming is a consistent feature of tumorigenesis [55, 62, 63].

Since Warburg first proposed aerobic glycolysis as a prerequisite for malignant transformation, mitochondrial dysfunction has been widely acknowledged as a hallmark of cancer [55, 64]. Carcinogenesis is driven by metabolic reprogramming to meet the bioenergetic and biosynthetic demands of cancer cells for their survival, development, and progression, while also modulating the tumor microenvironment (TME) [64, 65]. Mitochondrial bioenergetics, biosynthesis, ROS levels, and morphological dynamics have been linked to cellular stemness, including those of cancer cells [66, 67]. This suggests that mitochondria influence cancer stemness, which is characterized by the plastic cellular status of clonogenicity, self-renewal, and multilineage differentiation—key attributes underlying both cancer biology and evolution [10]. Additionally, mitochondrial DNA (mtDNA) mutations represent some of the most prevalent genetic alterations across all tumors and significantly impact metabolic homeostasis. Experimental evidence in mouse models demonstrates that mtDNA mutations at functionally significant sites, when present at high heteroplasmic levels, lead to defects in oxidative phosphorylation (OXPHOS). This can then facilitate metabolic reprogramming that promotes tumor proliferation [68]. Furthermore, intercellular transfer of intact, circular mtDNA via extracellular vehicles (EVs) derived from cancer cells induces metabolic and proliferative remodeling of adjacent epithelial cells in the TME. This process boosts proliferation, migration, and invasion of cancer cells by stimulating the secretion of transforming growth factor-β1 (TGF-β1), which in turn promotes epithelial-mesenchymal transition (EMT) and malignant transformation [69]. A recent, genome-wide Mendelian randomization analysis further identified mitochondrial dysfunction as a causal factor in multiple cancers, highlighting potential candidate genes involved in this process [70].

Similar to their role in cancer, mitochondria are also critical regulators of neuronal functions, governing axonal and dendritic development, synaptic activity, and metabolic support through neuron-glia interactions [71]. Alterations in mtDNA and mitochondrial function have been implicated in various NDDs [71, 72]. Dysregulation of ATP production, calcium homeostasis, and mitochondria-endoplasmic reticulum contact sites (MERCSs) has been observed in both NDD mouse models and patient-derived cells, including in AD and PD [71]. Mitochondrial dysfunction is a key determinant in the transition from a normal physiological state to neurodegeneration. Pathological protein aggregation, impaired ATP synthesis, and plaque formation–major hallmarks of NDDs– result from the interplay of genetic mutations and mitochondrial defects [72]. The mitochondrial cascade hypothesis of AD suggests that mitochondrial dysfunction precedes and drives the pathological accumulation of amyloid-beta (Aβ) and tau aggregates, exacerbating neurodegeneration. The interaction between amyloid precursor protein (APP) and Aβ with mitochondrial proteins disrupts mitophagy, leading to mitochondrial defects that contribute to disease progression [72]. PD is also primarily characterized by the degeneration and loss of dopaminergic neurons within the substantia nigra region and the abnormal accumulation of alpha-synuclein (α-synuclein, α-syn) protein [73]. Aggregation of α-syn oligomers, coupled with disrupted calcium homeostasis, induces mitochondrial membrane permeabilization and opening of the mitochondrial permeability transition pore (mPTP), leading to excessive ROS production, cytochrome C release, and neuronal apoptosis [72]. Autosomal recessive PD is associated with mutations in PTEN-induced kinase 1 (PINK1) and Parkin, resulting in mitochondrial respiratory deficits, increased neuronal vulnerability, oxidative stress, and impaired mitophagy activation [72]. Similarly, mitochondrial dysfunction manifests as mtDNA errors, oxidative stress, and calcium imbalance in Huntington’s disease (HD), showing more evidence of its crucial role in disease progression, as evidenced in both HD mouse models and human brain samples [72].

The nervous system, particularly the central nervous system (CNS), is also linked to cancer development [74]. Emerging epidemiologic and biological evidence suggests that the neural microenvironment interaction within TME actively contributes to the initiation and progression of various hematological and solid malignancies [75, 76]. Studies have demonstrated that mitochondria-to-nucleus coordination enhances the stemness of proximal cancer cells through neural signal-dependent activation of the cyclic AMP (cAMP)-ATF1 axis in the TME [77]. Notably, the high mitochondrial mass commonly observed in both cancer cells and CNS neurons underscores the crucial role of mitochondria in the pathogenesis of cancer and NDD [59, 78]. The inverse relationship between these two disease classes may be rooted in mitochondrial homeostasis, where shifts in mitochondrial function dictate cell fate decisions. Among the various mitochondrial regulatory mechanisms, mitophagy plays a decisive role in maintaining mitochondrial quality control (MQC). Disruptions in mitophagy may thus serve as a key mechanistic link between cancer and NDDs, contributing to their inverse epidemiological correlation [79, 80].

#### Mitophagy mechanism and pathways

Mitochondrial dysfunction disrupts energy production, metabolism, redox balance, and cell signaling, leading to cellular stress, tissue damage, and disease [81–84]. To maintain cellular homeostasis and functionality, damaged, dysfunctional, or superfluous mitochondria must be selectively removed through mitophagy, a specialized form of autophagy dedicated to MQC [83, 85, 86]. Mitophagy operates in synergy with mitochondrial biogenesis to regulate mitochondrial mass, function, quality, and metabolic modeling or reprogramming [59, 83, 86].

Mitophagy ensures mitochondrial quality and quantity control through two distinct but interconnected mechanisms: ubiquitin (Ub)-dependent and ubiquitin-independent pathways. These processes converge in the autophagy machinery, leading to the formation of mitophagosomes that encapsulate dysfunctional mitochondria for lysosomal degradation [83, 86–90].

#### Ub-dependent mitophagy

The Ub-dependent mitophagy pathway is primarily regulated by PINK1 and Parkin RBR E3 ubiquitin-protein ligase (Parkin, PRKN) [83, 86, 89, 90]. Under normal conditions, PINK1 is continuously imported into healthy mitochondria via the TOM/TIM complexes and cleaved by mitochondrial processing peptidases (MPP) and presenilin-associated rhomboid-like protein (PARL), leading to its proteasomal degradation [91]. However, upon mitochondrial stress (e.g., depolarization), PINK1 import is blocked, leading to its accumulation on the outer mitochondrial membrane (OMM). Stabilized PINK1 recruits and activates cytosolic Parkin, which ubiquitinates multiple OMM proteins, forming polyubiquitin chains that serve as signals for autophagy receptors, including sequestosome 1 (SQSTM1/p62), optineurin (OPTN), NBR1, TAX1BP1, and NDP52/CALCOCO2 [83, 92]. These receptors interact with ATG8-family proteins (e.g., LC3) on phagophores, facilitating autophagosome formation. The mitophagosomes then fuse with lysosomes for mitochondrial degradation and recycling [83, 92]. Beyond Parkin, additional E3 ubiquitin ligases such as SMAD-specific E3 ubiquitin protein ligase 1 (SMURF1), glycoprotein 78 (Gp78), mitochondrial ubiquitin ligase 1 (MUL1), seven in absentia homolog 1 (SIAH1), and Ariadne RBR E3 ubiquitin protein ligase 1 (ARIH1) also contribute to mitophagy regulation through ubiquitination of mitochondrial proteins [83].

#### Ub-independent mitophagy

The Ub-independent mitophagy pathway is mediated by mitophagy receptors, which bypass the need for ubiquitination by directly interacting with autophagy-related proteins. Key OMM-anchored receptors include BCL2-interacting protein 3 (BNIP3), BCL2-interacting protein 3-like (BNIP3L, NIX), BCL2-like 13 (BCL2L13), FUN14 domain-containing 1 (FUNDC1), and autophagy and beclin 1 regulator 1 (AMBRA1) [86, 92]. Additionally, inner mitochondrial membrane (IMM)-anchored receptors such as FKBP prolyl isomerase 8 (FKBP8) and Prohibitin 2 (PHB2), along with mitochondrial matrix proteins Nipsnap Homolog 1(NIPSNAP1) and Nipsnap Homolog 2 (NIPSNAP2), regulate this pathway. These receptors directly bind LC3, facilitating mitophagosome formation and mitochondrial degradation [86, 92].

#### Lipid-mediated mitophagy

Lipids also serve as mitophagy regulators. Under stress, cardiolipin externalizes from the inner to the outer mitochondrial membrane, where it facilitates the recruitment of autophagy-related proteins and fission machinery, thereby promoting mitophagy. Similarly, ceramide-LC3B (Microtubule-associated proteins 1A/1B light chain 3B) interactions have been implicated in lethal mitophagy instances in acute myeloid leukemia (AML), head and neck squamous cell carcinoma (HNSCC), and aged T cells [86].

#### Interplay between mitophagy pathways

While Ub-dependent and Ub-independent mitophagy operate via distinct mechanisms, they are functionally interconnected. AMBRA1 exemplifies this overlap, playing dual roles in both the PINK1/Parkin pathway and receptor-mediated mitophagy [86]. However, the precise mitochondrial signals triggering AMBRA1-mediated mitophagy remain largely unknown. Notably, AMBRA1 also regulates cyclin D degradation, linking mitophagy to cell cycle control, cancer development, and NDDs [93, 94]. The existence of parallel mitophagy pathways provides a robust fail-safe mechanism to eliminate damaged mitochondria under diverse cellular conditions [95]. The selective activation of either pathway is context-dependent, allowing cells to adapt to varying mitochondrial stressors and ensuring mitochondrial homeostasis [95].

Impaired PINK1/Parkin-mediated mitophagy is a key sign of several NDDs [96, 97]. Loss-of-function mutations in PINK1 or Parkin are directly linked to familial forms of PD, while reduced PINK1/Parkin activity is also observed in AD [97]. In amyotrophic lateral sclerosis (ALS), disrupted PINK1/Parkin signaling is associated with SOD1 and TDP-43 pathology [97]. In contrast, dysregulation of this pathway has been observed in various human cancers. Although Parkin plays a significant tumor-suppressive role via metabolic reprogramming, Parkin-mediated mitophagy appears to be dispensable for its tumor-suppressive function [98]. However, under specific stress conditions such as hypoxia or chemotherapy, the PINK1/Parkin signaling pathway can become reactivated in tumors, mitigating oxidative damage and promoting cancer cell survival [99].

In most NDDs, receptor-mediated mitophagy is less dominant than the PINK1/Parkin pathway, but it still plays a role under oxidative stress. For instance, BNIP3-mediated mitophagy induction in the nervous system increases organismal longevity and healthspan [100]. BNIP3 is further downregulated in cases of AD and PD [101, 102], and FUNDC1 exhibits dual-phase regulatory activity through phosphorylation status. Dephosphorylation, which activates mitophagy, is predominant in early AD, whereas hyperphosphorylation at Ser13 and Tyr18 is more prominent during late-stage AD. This modification not only influences Tau phosphorylation and Aβ metabolism but also exacerbates cognitive decline and promotes AD progression via multiple downstream signaling pathways [103]. Additionally, inhibition of FUNDC1 has demonstrated neuroprotective effects in PD [103]. Conversely, overexpression of FUNDC1 significantly improved locomotor function and extended survival in an ALS mouse model [104], with disrupted cardiolipin metabolism in both AD and PD potentially leading to mitochondrial synaptic dysfunction, oxidative stress, and neuroinflammation [105]. FUNDC1 further suppresses hepatocellular carcinoma (HCC) initiation by attenuating inflammasome activation and inflammatory responses in hepatocytes; however, its upregulation at later stages of tumor progression may support tumor growth [106]. Further evidence has been found as FUNDC1-mediated mitophagy has been implicated in resistance to chemotherapy [107]. The BNIP3 gene promoter also contains a hypoxia-responsive element: BNIP3 is broadly expressed in cancer cells and is involved in cancer growth in tissues under hypoxia or hypoxia-like conditions [108], where it facilitates tumor cell survival by promoting the removal of ROS-generating mitochondria through mitophagy [109]. Notably, BNIP3 may exert a dual role, as its overexpression has also been reported to induce cancer cell death under certain conditions [110].

Collectively, the distinct mitophagy mechanisms are differentially engaged in NDDs and cancers, with PINK1/Parkin signaling appearing to be more central to neurodegeneration, and receptor-mediated mitophagy pathways appearing to play a more prominent role in cancer. Whether they function as tumor promoters or suppressors appears to be highly dependent on the cancer subtype and the TME [98]. These mechanistic differences likely reflect the divergent metabolic demands and stress responses of neurons versus cancer cells, ultimately influencing disease onset and progression.

### Mitochondria, mitophagy and cancer

#### Insights into mitochondrial roles in cancer

Mitochondria are central to cellular metabolism and energy conversion, serving as hubs for signaling pathways that regulate cell function and fate. They import cytoplasmic substrates to fuel key metabolic pathways, including fatty acid oxidation (FAO), the tricarboxylic acid (TCA) cycle, the electron transport chain (ETC), and OXPHOS. Additionally, mitochondria synthesize essential biomolecules such as amino acids, lipids, nucleotides, heme, and iron-sulfur clusters. They also generate NADPH (nicotinamide adenine dinucleotide phosphate), supporting antioxidant defense mechanisms to maintain cellular redox balance [59, 111]. Further, mitochondria dynamically coordinate energy production and distribution in response to caloric and oxygen availability while fulfilling cellular demands for maintenance and proliferation. In many cancer cells, metabolic reprogramming shifts energy production from OXPHOS, which optimizes ATP generation in quiescent and differentiated cells, to aerobic glycolysis (the'Warburg effect'). This shift prioritizes biosynthesis over energy efficiency, supplying macromolecular precursors necessary for rapid proliferation and enhanced mitochondrial biogenesis [59, 111]. However, not all tumors rely on aerobic glycolysis, and mitochondrial respiration defects are neither the primary driver of the Warburg effect nor a standardized feature during tumor evolution [59].

Mitochondria regulate numerous cellular and metabolic functions, and disruptions in their normal activity have been implicated in various human malignancies. Cancer development is driven by oncogene activation, tumor suppressor gene inactivation, and malignant alterations across multiple omics layers, including the genome, epigenome, transcriptome, epitranscriptome, proteome, and metabolome. These molecular changes are key causes of cancer, driving the progression from normal cellular states to pre-malignant stages and ultimately to fully developed neoplasia [11, 112–114]. Additionally, mitochondria interact with other intracellular organelles both directly and indirectly, influencing cellular homeostasis and contributing to cancer pathogenesis [56, 59, 86, 87, 115–117].

### Mitochondria-Nucleus crosstalk in cancer

#### Basis of mitochondria-nucleus crosstalk in cancer

Reciprocal Nuclear-Mitochondrial communication plays a crucial role in cellular homeostasis and cancer development**.** Physical contact sites known as mitochondria-associated nucleus (NAM) structures facilitate this overlap, enabling mitochondrial regulation of nuclear processes**.** A molecular bridge between translocator protein (TSPO)-positive mitochondria and the nucleus facilitates this communication [118]. KRAS mutation (KRAS*)—the key driver mutation in pancreatic ductal adenocarcinoma (PDAC) and non-small cell lung cancer (NSCL)—also plays a secondary role in colorectal carcinoma (CRC) progression [114]. KRAS* induces BNIP3L expression, activating a selective mitophagy program that limits glucose flux to mitochondria while enhancing redox capacity. The induction of BNIP3L and suppression of mitochondrial ROS (mtROS) by KRAS* are critical for early pancreatic cancer development, as impaired mitophagy delays tumor progression [119, 120]. In pancreatic tissue-specific branched-chain amino acid transaminase 2 (Bcat2) knockout models, pancreatic intraepithelial neoplasia (PanIN) progression is suppressed in *LSL-Kras*^G12D/+^; *Pdx1-Cre* (KC) mice. Functionally, BCAT2 enhances branched-chain amino acid (BCAA) uptake, supporting mitochondrial respiration and BCAA catabolism. Inhibiting BCAT2 reduces PanIN formation in KC mice, underscoring the critical role of mitochondrial BCAT2-mediated BCAA metabolism in KRAS-driven PDAC development [121]. Meanwhile, Retrograde signaling from mitochondria can modulate nuclear gene expression, influencing cell behavior and enabling adaptation to mitochondrial stress. This process contributes to tumor initiation, survival, metastasis, and therapy resistance. Nuclear ribonucleoprotein-A2 (hnRNP A2) plays a pivotal role in this pathway by activating target genes through the recruitment of DNA-binding factors such as NFκB (c-Rel/p50), C/EBPδ, CREB, and NFATc, or by stabilizing the enhanceosome complex for mitochondrial respiratory stress-responsive gene expression [122, 123].

This intercellular movement of mitochondria (mitochondrial transfer, MT) has emerged as a novel and significant process in cancer biology, contributing to tumor growth, metastasis, and therapeutic resistance by reshaping the metabolic landscape of TME cells [124–127]. This MT restores mitochondrial function, cellular respiration, and tumor-forming capacity [127, 128]. Beyond restoring bioenergetics, MT also has the potential to reprogram the metabolic state of recipient cells, further influencing tumor progression [129–138]. This is due to MT’s direct link with increased cell cycle activity and poor clinical outcomes. Further validation in clinical samples is needed, though TNT-mediated MT may be a key immune evasion mechanism in vivo [139]. Damaged mitochondria, along with the release of N-formyl peptides and mtDNA, can act as damage-associated molecular patterns (DAMPs), triggering innate immune activation [140–145]. Mitophagy-related protein Parkin can inhibit EV-driven processes by promoting the degradation of damaged mitochondrial content through lysosomal pathways [146]. Mitocytosis is a mitochondrial quality control (MQC) process by which damaged mitochondria are transported into migrasomes and expelled by migrating cells [147, 148]. Mitocytosis is a mechanism by which cells alleviate the burden of damaged mitochondria, thereby maintaining MQC. This process is intrinsically linked to cellular migration, highlighting its important role in preserving cell health and disease progression [148]. Meanwhile, the transfer of mitochondria via EVs has also been implicated in the development of drug resistance in cancer cells [149, 150]. Tumor cells lacking mtDNA only form tumors after acquiring mtDNA from host cells [127]. Further, single-cell RNA sequencing (scRNA-seq) data from human cancers reveal that mtDNA transfer from T cells to cancer show how MT may be a key immune evasion mechanism in vivo [139].

#### Mitochondria dynamics

The regulation of mitochondrial dynamics and ultrastructure is orchestrated by a network of mitochondria-shaping proteins that govern key processes such as fusion, fission, and cristae remodeling [151]. Deregulation of mitochondrial dynamics has been implicated in tumorigenesis and immune infiltration, with differential expression of mitochondrial dynamics-related proteins observed between tumor tissues and their adjacent non-tumor counterparts [152–156]. Moreover, these alterations may serve as potential predictive biomarkers for patient prognosis [156–161].

Aberrant mitochondrial dynamics also contribute to tumor proliferation, drug resistance, metastasis, and the acquisition of stem-like properties [158, 162]. Further, DRP1-mediated mitochondrial fission plays a crucial role in regulating fatty acid (FA) and glucose metabolism, thereby promoting CRC cell proliferation, invasion, and migration [163, 164]. DRP1 has been shown to enhance tumor-associated macrophage (TAM) infiltration and T cell immune-surveillance [165, 166]. Mitochondrial fission regulator 2 (MTFR2), ADP-ribosylation factor 1 (ARF1), and the ovarian tumor-associated protease deubiquitinase 6 A (OTUD6A) are also significantly overexpressed in CRC tissues, suggesting their potential roles in mitochondrial dynamics and cancer progression [167–169]. Overall, mitochondrial dynamics and mitophagy are conserved, interrelated processes that work together to maintain mitochondrial health by isolating and removing damaged mitochondria [170]. Mitochondria maintain internal homeostasis through tightly regulated processes including fission, fusion, mitophagy, mitochondrial transport, and biogenesis [171]. Fusion allows damaged mitochondria to merge with healthy ones, facilitating functional complementation and repair. Conversely, fission segregates dysfunctional mitochondrial fragments, which are then targeted for degradation via mitophagy [172]. This quality control system ensures optimal mitochondrial function [172]. Disruption of the balance between mitochondrial fission/fusion and mitophagy can impair mitochondrial integrity and contribute to the pathogenesis of various NDDs [173]. Since mitochondrial fission factors DNA1L/DRP1 are dispensable for receptor-mediated mitophagy [174], mitochondrial fission may be a prerequisite for mitophagy [175]. This occurs partially because of fission shedding damaged parts into smaller mitochondria destined for mitophagy [176]. The mitophagy receptor FUNDC1 also interacts with DRP1 to promote fission, facilitating the removal of damaged mitochondria and maintaining cellular homeostasis [177]. Overall, mitochondrial fission/fusion and mitophagy are reciprocally regulated and functionally interconnected, and their cooperative role in maintaining neuronal health is paramount [178].

#### mtDNA variation and numtogenesis

Somatic alterations in mtDNA copy number, mutations in mitochondrial genes, and changes in the D-loop region have also been implicated in degenerative diseases, cancer, and aging [179, 180]. Variants in mtDNA are associated with differential tumorigenicity [181], yet the mechanisms by which mtDNA mutations contribute to tumorigenesis remain poorly understood. Notably, certain mtDNA mutations can influence ROS production and redox homeostasis, thereby promoting tumor cell growth [180, 182–184]. Additionally, it has been proposed that alterations in both mtDNA and nuclear DNA (nDNA) encoding mitochondrial genes may affect mitochondrial metabolite production, which, in turn, could modify the epigenome and regulate nDNA gene expression [180, 184–186]. Beyond their metabolic and oncogenic roles, mtDNA variation also influences immune function through distinct mechanisms. They modulate the adaptive immune system by altering the suppressive capacity of regulatory T (Treg) cells and impact the innate immune system by enhancing the aggressiveness of conventional T (Tconv) cells. While inhibition of mitochondrial ROS (mtROS) may mitigate their mitogenic effects on tumor growth, this reduction could suppress both the adaptive and innate immune responses, potentially impairing immune-mediated tumor rejection [187].

Numtogenesis is the biological process by which mtDNA is transferred into the nuclear genome. The resulting integrated mtDNA fragments within the nuclear genome are referred to as nuclear mitochondrial DNA (NUMTs) [188, 189], which have the potential to induce mutations and/or activate proto-oncogenes [188]. The active transfer of mtDNA sequences to the nuclear genome contributes to the intricate network of mito-nuclear communication mechanisms that influence human health [190, 191]. Notably, NUMTs have been detected at a higher frequency in the tumor tissues compared to normal tissues [188, 192]. An increase in numtogenesis has been particularly observed in CRCs, where the abundance of NUMTs in malignant cells correlates with poorer patient prognosis and survival. Collectively, current evidence suggests that increased numtogenesis may contribute to cancer risk and prognosis. However, further research is needed to improve NUMT detection methods, elucidate the molecular mechanisms governing numtogenesis, and establish causal relationships between NUMT insertions and tumorigenesis [189]. Although direct evidence linking mitophagy and numtogenesis is currently lacking, it is plausible that effective mitophagy mitigates numtogenesis by promoting the clearance of damaged mitochondria and limiting the release of mtDNA into the cytoplasm and nucleus. On the other hand, impaired mitophagy may lead to the accumulation of dysfunctional mitochondria, increasing the likelihood of mtDNA leakage and subsequent integration into the nuclear genome, thereby contributing to NUMT formation and genomic instability.

#### mtROS

Mitochondria are a major source of ROS in mammalian cells [193, 194]. Cellular proteins, particularly those containing thiol residues, are key targets of ROS signaling [193]. These oxidative modifications alter protein enzymatic activity, conformation, and function, thereby influencing multiple biological processes [195, 196]. Physiological fluctuations in ROS play a crucial role in cellular signaling, but excessive ROS can cause oxidative damage to proteins, nucleic acids, and lipids [197]. At smaller physiological levels, ROS can also regulate essential signaling pathways such as mitogen-activated protein kinases (MAPK), phosphoinositide 3-kinase (PI3K)/protein kinase B (PKB, Akt), apoptosis signal-regulating kinase 1 (ASK1), and NAD + -dependent deacetylase Sirtuin 1 (SIRT1)-Forkhead box class O (FoxO)/TRP53 transcriptions [196, 198, 199].

ROS primarily induces DNA mutagenesis/damage and protein contributions to carcinogenesis of nucleobases [197, 200–204]. Elevated ROS levels not only influence metabolic pathways [205, 206]. This metabolic shift aids cellular adaptation and drives metabolic reprogramming, potentially leading to phenotypic changes associated with tumorigenesis [207]. Before acinar-to-ductal metaplasia (ADM)—a precursor to PDAC [208, 209]—develops, oncogenic KRAS elevates acetyl coenzyme A (CoA) and ROS levels, with inhibition of these metabolic alterations preventing ADM formation [208–210]. Similarly, metabolic changes identified in premalignant colonic polyps persist in adenocarcinomas in both humans and mice [211, 212]. In liver cancer models, MYC-driven metabolic reprogramming precedes tumorigenesis, with early-stage premalignant hepatocytes favoring pyruvate-to-alanine conversion, whereas later-stage malignant lesions preferentially convert pyruvate to lactate [213]. Tumorigenic mutations in KRAS, TRP53, MYC, and other oncogenes frequently reprogram metabolic pathways related to energy production (including the Warburg effect), macromolecular synthesis, and redox balance [208]. The tumor and its TME cooperate to regulate metabolic heterogeneity, driving proliferation, invasion, metastasis, and therapy resistance [208]. Metabolic alterations and ROS production are intertwined in cancer cells [214], with ROS promoting metastasis in some models through metabolic reprogramming and signaling functions [215–217].

### Mitophagy and cancer

#### Implications of mitophagy in cancer

Mitochondria-mediated metabolic reprogramming is a hallmark of cancer [18, 19, 31, 117], with tumorigenesis driven by metabolic alterations in response to tumor-promoting etiological factors [19]. Mitochondrial dysfunction is closely linked to several classical cancer characteristics, as oncogenic signaling pathways modulate mitochondrial biology to facilitate cellular transformation [31, 117]. Mitochondria contribute to cancer progression and metastasis by regulating metabolic and genetic responses to dynamic microenvironmental cues [218, 219]. Mitophagy also plays a crucial role in maintaining mitochondrial adaptation to these cues and is essential at all stages of cancer biology, due to its influences on metabolic reprogramming, transformation, and tumor progression [208, 211, 212, 220–223]. However, mitophagy remains complex and not fully understood, exhibiting a dual role in tumorigenesis-either promoting or suppressing cancer progression depending on the intrinsic properties of cancer cells and the characteristics of their TME [83, 224–228].

In early malignant transformation, mitophagy acts as a tumor-suppressor by removing damaged mitochondria (major sources of ROS) [229], thereby reducing oxidative stress and preventing genomic instability [230]. This function is critical for inhibiting tumor initiation. However, in established tumors, mitophagy can become pro-tumorigenic by promoting cancer cell survival under hypoxia and facilitating invasion and metastasis [231, 232]. However, it’s important to note that mitophagy may also suppress tumor progression by limiting the proliferative outgrowth of dormant disseminated tumor cells (DTCs) at metastatic sites [233]. In breast cancer, mitophagy supports dormant DTCs by maintaining a healthy mitochondrial network essential for OXPHOS and preventing ROS accumulation, thereby empowering dormant DTC survival in the lung [233, 234]. Furthermore, PARK2 exhibits tumor-suppressive properties, with mutations identified in human GBM, colon cancer, and lung cancer [235]. Additionally, PINK1 and PARK2 suppressed pancreatic tumorigenesis [236], reinforcing their tumor suppression. The mitophagy receptor BNIP3 also functions as a tumor suppressor in mammary and pancreatic tumorigenesis [237, 238], illustrating mitophagy’s crucial role in cancer regulation.

#### Mitophagy and immune surveillance

Immune cells rely on mitochondria to meet their dynamic metabolic demands during the immune response. Mitophagy regulates mitochondrial adaptation and metabolic remodeling, ensuring MQC and functional optimization; however, its role in adaptive immunity remains unclear. The metabolic reprogramming mechanisms driven by tumor-immune cell interactions have been extensively reviewed elsewhere [239–241]. Here, we focus on recent findings highlighting mitophagy’s influence on immune cells within TME.

Tumor cells actively shape the metabolic landscape of the TME through nutrient consumption and metabolite production, influencing the metabolic programming and adaptation of immune cells [239]. Mitophagy is essential for this reprogramming [80], as local metabolic changes derived from metabolic reprogramming modulate immune cell survival and function, ultimately favoring immune escape and tumor progression [239]. Further, mitophagy’s crucial role in immune regulation and surveillance is conducted through multiple mechanisms [242]. First, it regulates the development and differentiation of immune cells, including T cells, natural killer (NK) cells, and macrophages [242]. Additionally, mitophagy influences mitochondrial antigen presentation (mtAP) and modulates cytokine secretion [242]. By maintaining MQC, mitophagy supports immune cell function and metabolic adaptation. In T cells, it governs activation and differentiation, while in NK cells, it is essential for cytotoxic activity [242, 243]. In macrophages, mitophagy contributes to polarization and immune homeostasis. Moreover, mitophagy regulates inflammation by controlling mtROS and DAMPs, thereby influencing cytokine production [242].

Mitophagy is also a key factor in the maintenance of T cell survival and homeostasis [243, 244], with metabolic remodeling occurring at all stages of CD4⁺ and CD8⁺ T cell activation and differentiation. The intricate interplay between metabolic pathways, epigenetic modifications, and transcription factor expression plays a pivotal role in regulating T helper (Th) cell plasticity, influencing their function and fate [245]. Regulators of mitophagy, such as Parkin and NIX, are upregulated in response to interleukin-15 (IL-15) to support memory T cell formation, suggesting that the mitophagy machinery is crucial for orchestrating survival and metabolic dynamics essential for the development and maintenance of memory T cells [246]. Urolithin A (UA), a naturally occurring compound that stimulates mitophagy, has been shown to enhance anti-tumor CD8⁺ T cell immunity in vivo. UA-induced memory T stem cell formation relies on PINK1-mediated mitophagy, which facilitates the release of phosphoglycerate mutase family member 5 (PGAM5) into the cytoplasm. Cytosolic PGAM5 subsequently dephosphorylates β-catenin, activating the Wnt signaling pathway and promoting mitochondrial biogenesis [247].

Upon persistent antigen stimulation, T cells upregulate inhibitory molecules such as PD-1 and cytotoxic T-lymphocyte-associated protein 4 (CTLA-4). The engagement of these inhibitory receptors, whether on anti-tumor T cells or Tregs, leads to metabolic reprogramming, including alterations in fatty acid oxidation (FAO) and glycolysis, which modulate T cell function and persistence [245, 248]. Decreased mitophagy activity impairs the metabolic fitness and antitumor efficacy of CD8^+^ tumor-infiltrating T lymphocytes (TILs), manifesting characteristics of terminal exhaustion [249]. For example, intratumoral T cells display reduced mitophagy, resulting in the accumulation of depolarized mitochondria and increased mtROS. This mitochondrial dysfunction accelerates their differentiation into a terminally exhausted phenotype, thereby compromising their persistence and anti-tumor functionality [245, 249–252].

Additionally, the immunosuppressive metabolic TME is known to profoundly influence the anti-tumor immune response. Contributing factors include tumor-derived cytokines, as well as the hypoxic, acidic, and nutrient-deprived conditions within the TME, which collectively impair the function of T cells and NK cells [245, 253]. Hypoxia promotes the expression of immunosuppressive molecules such as vascular endothelial growth factor (VEGF), TGF-β, IL-10, and PD-L1 and induces the recruitment of cancer-associated fibroblasts (CAF) to facilitate the infiltration of myeloid-derived suppressor cells (MDSCs), Tregs, and type 2 tumor-associated macrophages (TAMs) via hypoxia-induced metabolic alterations in the tumor and TME [254, 255]. Tumors deplete critical nutrients such as glucose, glutamine, arginine, aspartate, asparagine, and serine, thereby starving effector immune cells while promoting the expansion of immunosuppressive cell populations. These include MDSCs and Tregs [245, 256]. T helper 1 (Th1), T helper 2 (Th2), and T helper 17 (Th17) effector cells, which primarily rely on glycolysis for their energy needs, whereas immunosuppressive Tregs predominantly depend on lipid oxidation to meet their metabolic demands [245].

Mitophagy supports the development of robust NK cell memory in viral infection models by eliminating ROS and dysfunctional mitochondria through BNIP3- and BNIP3L-mediated signaling pathways [257]. Fragmentation of mitochondria in tumor-infiltrating NK cells has been linked to diminished cytotoxic function and NK cell depletion, which in turn facilitates tumor evasion from NK cell-mediated immune surveillance [258]. Additionally, cyclic fasting enhances NK cell anti-tumor immunity through metabolic reprogramming [259]. Recent analysis of a single-cell transcriptome dataset revealed that mitophagy-related genes (MRGs) exhibit significantly higher activity in TME of oral cancer compared to other cell types [260].

Given that cancer cells can control macrophage recruitment and polarization within TME, shifting macrophages from an M1 phenotype with anti-tumor properties to an M2-polarized macrophage (also known as TAM) promotes tumorigenesis [261]. Notably, mitophagy acts as a key factor in M1/M2 differentiation. AMP-activated protein kinase (AMPK) is activated, triggering FAO, which fuels OXPHOS [262]. ROS production has been shown to activate AMPK, inducing mitophagy and promoting M2 macrophage polarization in monocytes [263]. M2 macrophages secrete immunosuppressive and pro-tumorigenic factors, including IL-10, epidermal growth factor (EGF), VEGF, matrix metalloproteinases (MMPs), deoxypyridinoline (DPD), platelet-derived growth factor (PDGF), and TGF-β, thereby facilitating immune suppression and tumor progression [255].

CAFs are the predominant stromal cell type in solid cancer TME. CAFs evolve alongside tumor progression, acquiring a catabolic phenotype through metabolic reprogramming [264]. The differentiation of CAFs is crucial for cancer initiation and progression, and it is regulated by signaling pathways induced by tumor cell-derived TGF-β, EGF, PDGFα, PDGFβ, basic fibroblast growth factor (bFGF, also known as FGF2), interleukin 6 (IL-6), and interleukin 1β (IL-1β) [264]. Oxidative stress in CAFs drives mitophagy and aerobic glycolysis-based metabolic reprogramming, known as the reverse Warburg effect [265, 266]. In a breast cancer model, integrin β4 (ITGB4) protein derived from triple-negative breast cancer (TNBC) cells promotes glycolysis in CAFs through BNIP3L-dependent mitophagy. This process is initiated following the transfer of ITGB4-containing exosomes into CAFs [267]. In an orthotopic PDAC mouse model, inhibiting mitophagy by targeting Parkin in CAFs impairs tumor growth in vivo. Autophagy deficiency in CAFs enhances proline biosynthesis through mitophagy-mediated regulation of NAD kinase 2. Additionally, mitophagy in CAFs facilitates collagen secretion by modulating proline synthesis, thereby promoting CAF activation and supporting tumor progression in vivo [268].

#### Mitophagy and cancer stem cells (CSCs)

Mitophagy functions as an adaptive response to pathophysiological stressors, playing an essential role in MQC. It preserves mitochondrial function, limits excessive ROS production, and optimizes the utilization of limited metabolites and oxygen within TME [224]. CSCs rely on mitophagy to maintain mitochondrial health, ensuring optimal function and energy production necessary for their self-renewal, pluripotency, and tumorigenic potential [269–271]. CSCs depend on mitophagy for metabolic reprogramming, allowing them to adapt to stressful microenvironments and sustain their survival and functionality [270, 271]. Even slight variations in ROS levels can significantly impact stem cell fate, given that ROS signaling is pivotal in regulating CSCs’ homeostasis and lineage commitment [272]. The selective removal of damaged mitochondria via mitophagy prevents excessive ROS accumulation, thereby preserving CSCs’ function and maintaining their stemness. Studies indicate that BNIP3/BNIP3L-dependent mitophagy supports CSCs’ anchorage-independent propagation, such as mammosphere formation, and is associated with enhanced metabolic activity, increased ATP production, and elevated antioxidant capacity. This process contributes to greater CSC proliferation and migratory potential [273]. Additionally, mitochondrial fission 1 protein (FIS1) and AMPK are key regulators of mitophagy in leukemic stem cells (LSCs). Genetic inhibition of both FIS1 and AMPK impairs mitophagy, resulting in cell cycle arrest and loss of stemness in LSCs [271].

Mitophagy regulates hepatic cancer stemness through NANOG activation by facilitating the degradation of mitochondrial phosphorylated p53, a process mediated by PINK1 [274]. Wild-type p53 suppresses the expression of key liver CSCs markers, including CD44, c-Myc, NANOG, SOX2, and OCT4. In contrast, p53 mutations abolish this suppressive effect, resulting in enhanced CSCs marker expression and increased resistance to radiotherapy and chemotherapy [275]. Mitophagy is instrumental in regulating the properties of CSCs, which are subpopulations of tumor cells with self-renewal and differentiation abilities, contributing to tumor initiation, progression, and anti-therapeutic adaptation.

Mitophagy is further activated in cancer cachexia and plays a crucial role in skeletal muscle atrophy through mitophagy-mediated inflammation, ROS accumulation, and mitochondrial degeneration [276, 277]. This process is characterized by an increase in mitophagy-related proteins such as BNIP3 and PINK1, along with reduced levels of key regulators of mitochondrial dynamics, including mitofusin 2 (MFN2), OPA1, FIS1, and DRP1 [276, 278]. These changes contribute to mitochondrial dysfunction and muscle wasting associated with cancer cachexia.

### Mitochondria, mitophagy and NDDs

#### Implication of mitochondria in the brain

The human brain contains billions of distinct cells, and its function is highly energy-demanding, with neurons being the most energy-consuming cell type. At rest, the brain accounts for approximately 20% of the body's total energy consumption, despite constituting only 2% of the body’s overall weight [279, 280]. The brain's limited energy reserves can rapidly impair cognitive function, particularly since nerve terminals do not store sufficient ATP molecules and must continuously synthesize them during periods of neural activity [281]. Non-neuronal cells, including astrocytes and microglia, play a pivotal role in regulating the functional interactions between specific neuronal subpopulations and their corresponding metabolic processes. For example, the “astrocyte-neuron lactate shuttle” is a critical metabolic pathway in which astrocytes act as a central metabolic hub in the brain [282, 283]. Overall, brain activity depends on dynamic and coordinated metabolic processes, highlighting the critical importance of mitochondrial health.

Mitochondria are indispensable for brain functions specifically because of their contributions to functions such as bioenergetics, biosynthesis, redox balance, calcium homeostasis, apoptosis regulation, and signal transduction [279, 280, 284–287]. The majority of ATP in the brain is generated within mitochondria through OXPHOS. The ATP produced by mitochondria is essential for neuronal activity, supporting various energy-demanding processes. The regulation of ATP metabolism, including both its production and utilization, is vital for cerebral bioenergetics, brain function, and the pathophysiology of NDDs [288]. Beyond ATP synthesis, mitochondria serve as biosynthetic hubs for the synthesis of crucial biomolecules, including nucleotides, fatty acids, cholesterol, amino acids, glucose, and heme. These metabolites are pivotal in the formation of macromolecules such as lipids, proteins, DNA, and RNA, which are critical for cellular function. Moreover, mitochondria generate metabolic by-products, such as ROS and ammonia, and possess specialized mechanisms for their clearance or utilization, thereby preserving cellular homeostasis [285]. Heme, synthesized within mitochondria, plays an essential role in neuronal survival, growth, and repair. As both a signaling molecule and a modulator of cellular stress responses, heme interacts with various molecular pathways to regulate neuronal function. Its deficiency has been linked to NDDs, as it can alter the processing of APP and α-syn and disrupt neuronal function in AD and PD [289].

Growing evidence highlights the essential and distinctive role of mitochondria in synaptic transmission. Mitochondria participate in multiple aspects of neurotransmission, including neurotransmitter synthesis and storage, synaptic vesicle (SV) trafficking, neurotransmitter release at presynaptic terminals, and SV recycling. It is well established that mitochondria primarily facilitate synaptic transmission by maintaining calcium homeostasis, supplying energy, regulating ROS production, and synthesizing essential intermediates or final products required for neurotransmitter production [290].

Calcium (Ca^2+^), as a secondary messenger, is fundamental to normal neuronal activity and brain function, regulating the synthesis and secretion of neurotransmitters [291]. Intracellular and mitochondrial calcium homeostasis is synergistically maintained through the intricate interplay between ER and mitochondria, facilitated by both physical contacts and complex signaling pathways. This crosstalk is primarily mediated by mitochondria-associated membranes (MAMs), specialized structures that establish direct ER-mitochondria communication [291]. These structures play a crucial role in regulating Ca^2^⁺ homeostasis through various signaling mechanisms: The IP₃R-VDAC-MCU Pathway facilitates Ca^2^⁺ transfer from the ER to mitochondria via inositol 1,4,5-trisphosphate receptors (IP₃Rs) on the ER membrane, voltage-dependent anion channels (VDACs) on OMM, and MCU on IMM. The interaction between these components at MAMs is essential for Ca^2^⁺ signaling and homeostasis [291, 292]. Other key signaling pathways include the Sigma-1 Receptor (Sig1R) Pathway [291], the AKT-mTOR pathway [293], the PINK1-Parkin pathway [294], and the PERK-eIF2α-CHOP Pathway [295]. Calcium homeostasis is critical for normal mitochondrial function, influencing ATP production, apoptosis regulation, ROS production, and metabolic regulation of FA oxidation and the urea cycle [296–300].

Unlike extrinsic apoptosis, which is triggered by death receptor signaling, mitochondria serve as the central regulators of intrinsic (mitochondrial) apoptosis, orchestrating the balance between cell survival and programmed cell death [301]. The key mechanisms of mitochondrial apoptosis include mitochondrial outer membrane permeabilization (MOMP), a process governed by coordinated actions of pro-apoptotic proteins (Bax, Bak, Bid, and Bim) and counteracted by anti-apoptotic proteins (Bcl-2, Bcl-xL, and Mcl-1) [57, 302]. Following MOMP, cytochrome c is released into the cytosol, where it binds to apoptotic protease-activating factor-1 (Apaf-1) and ATP, forming the apoptosome. The complex activates caspase-9, which subsequently triggers the activation of caspase-3 and caspase-7, leading to cellular dismantling and apoptosis execution [84, 302]. Additionally, mPTP opening results in mitochondrial swelling and rupture, thereby amplifying apoptotic signaling [84, 302]. The regulation of mPTP opening is modulated by factors such as calcium overload, oxidative stress, and adenine nucleotide levels [303]. Furthermore, caspase-independent mitochondrial apoptosis is mediated by apoptosis-inducing factor (AIF) and endonuclease G (EndoG), which translocate to the nucleus and trigger chromatin condensation and DNA fragmentation [302, 304, 305]. Notably, mitochondria maintain a close functional interplay with the ER, contributing to the regulation and activation of mitochondrial apoptotic pathways [306].

Mitochondria regulate multiple signaling pathways that integrate metabolic, apoptotic, and stress responses. The intrinsic (mitochondrial) apoptosis pathway and MAM signaling in ER-mitochondria crosstalk, as briefly outlined above, are pivotal in determining cell survival and death [297, 307]. The mitochondrial unfolded protein response (UPR^mt^) is essential for maintaining mitochondrial protein homeostasis (proteostasis) under stress conditions. This adaptive mechanism is orchestrated by a complex signaling network involving activating transcription factor 6 (ATF6), inositol-requiring kinase 1 (IRE1), and double-stranded RNA-activated protein kinase-like PERK [308]. The AMPK-mTOR axis governs mitochondrial biogenesis and mitophagy, thereby controlling energy homeostasis and facilitating adaptation to nutrient availability. Activation of AMPK upregulates PGC-1α, thereby enhancing mitochondrial biogenesis, while concurrently inhibiting mTOR to promote mitophagy [309, 310]. The PI3K-Akt pathway supports cellular metabolism, mitochondrial function, and survival under growth-promoting conditions. Through PI3K-Akt-mTORC1 signaling, this pathway augments glycolysis, mitochondrial metabolism, cell growth, and biosynthetic processes [311, 312]. Additionally, redox signaling and ROS regulation are fundamental in modulating oxidative stress and maintaining redox homeostasis in response to metabolic demands [313].

NDDs encompass a diverse group of disorders characterized by the progressive and selective degeneration of specific neuronal populations, resulting in functional impairments. Examples of major NDDs we’ll explore include AD, PD, HD, and ALS. Among the various pathological mechanisms implicated in NDDs, mitochondrial dysfunction has emerged as a central contributor to disease progression [24]**.** In this context, we will now explore the pivotal role of mitochondrial dysfunction and mitophagy in the brain, and in the pathogenesis of NDDs.

#### Mitophagy in the brain

Mitophagy profiling in mice has revealed distinct alterations across various brain cell types, including dopaminergic neurons, Purkinje cells, astrocytes, microglia, and interneurons, as well as within specific brain subregions [314, 315]. As the primary MQC mechanism in neurons, mitophagy plays a crucial role in preserving neuronal health by efficiently eliminating damaged mitochondria, thereby mitigating oxidative stress, preventing mitochondrial dysfunctions, and reducing the risk of neurodegeneration [314].

While basal mitophagy is essential for maintaining neuronal homeostasis, its activation can be upregulated in response to various pathophysiological stimuli [316]. Mitochondrial removal presents a complex cellular process, as most mitochondria reside in distal neuronal compartments, whereas lysosomes, responsible for mitophagy degradation, are predominantly concentrated in the neuronal cell body. This spatial separation necessitates efficient transport mechanisms to ensure proper mitochondrial clearance [317]. Both in vitro and in vivo studies consistently indicate that neuronal mitophagy primarily occurs in the soma, a selective process that confines damaged mitochondria to the cell body, thereby mitigating mitochondrial dysfunction in distal axons [316]. However, due to their polarized morphology of neurons, characterized by long axons and extensive terminal branching, damaged axonal and presynaptic mitochondria are either transported retrogradely for degradation or local mitophagy [317, 318]. The PINK1-Parkin mitophagy pathway is conserved in both neuronal and non-neuronal cells, as demonstrated by in vitro induction experiments. Upon focal acute damage to axonal mitochondria, the PINK1–Parkin pathway is activated, triggering local mitophagy [319]. However, Parkin translocation in neurons appears to be significantly slower, suggesting that mitochondria may undergo mitophagy through more distinct regulatory mechanisms compared to non-neuronal cells [317]. Parkin-mediated mitophagy is upregulated in neurons following OXPHOS stimulation [320]. The mild dopaminergic loss phenotypes observed in PINK1 and Parkin knockout (KO) mice suggest the presence of compensatory MQC mechanisms that help maintain cellular homeostasis in the absence of PINK1–Parkin–mediated mitophagy [317]. Additionally, nerve growth factor (NGF) plays a critical role in regulating neuronal differentiation by stimulating the dynamic cycle of mitochondrial fusion, fission, and selective mitophagy. This coordinated remodeling of the mitochondrial network enhances mitochondrial quality control and bioenergetic capacity, ultimately supporting neuronal maturation and function [321].

Synaptic transmission relies on a substantial local energy supply, the maintenance of presynaptic energy homeostasis, and calcium buffering, all of which are supported by a healthy population of axonal mitochondria through local mitochondrial biogenesis, maintenance, and mitophagy-mediated MQC mechanisms [318]. Wdfy3(WD Repeat And FYVE Domain Containing 3) haploinsufficiency results in reduced mitophagy, accumulation of morphologically altered mitochondria, decreased synaptic density, and impaired synaptic plasticity [322, 323]. Synaptic transmission constitutes the fundamental process of communication, forming the basis of neural functions [324, 325]. Neuronal mitophagy is crucial for synaptic plasticity, which underlies synaptic transmission. Dynamic alterations in mitochondrial metabolism and proteome remodeling across distinct neuronal states may differentially activate mitophagy pathways, thereby influencing synaptic function and plasticity.

PINK1-mediated ubiquitin phosphorylation predominantly occurs in astrocytes, though it is also observed in other cell types, including neurons, microglia, and oligodendrocyte progenitor cells [326]. The differential mechanisms of mitophagy between neuronal and non-neuronal cells remain to be fully discovered. In dopaminergic neurons, damaged mitochondria accumulate within spheroid structures containing immature mitophagosomes, which are subsequently transferred to astrocytes for degradation through a Parkin-dependent transmitophagy process [61, 314, 327]. This mechanism underscores the critical role of astrocytes in maintaining neuronal mitochondrial health [61, 314]. Microglia, the resident innate immune cells of the brain, serve as the first line of defense in neuroimmune responses and play essential roles in maintaining neuronal function and integrity [328]. Enhancing microglial mitophagy not only facilitates the resolution of neuroinflammation but also improves phagocytosis and the clearance of pathological proteins associated with NDDs, including Aβ plaques, mutant huntingtin protein (mHTT) aggregates, and TAR DNA Binding Protein 43 kDa (TDP-43) protein inclusions [329–332]. Furthermore, tunneling nanotube (TNT)-mediated communication between neuronal and microglial cells enables the bidirectional transfer of α-syn and mitochondria. Through the transfer of healthy mitochondria, microglia can rescue neurons experiencing oxidative stress and dysfunction induced by toxic aggregates of α-syn and tau [333, 334]. Thus, microglia play an indispensable role in maintaining brain health and neuron function.

Oligodendrocyte precursor cells (OPCs) differentiate into oligodendrocytes, which are responsible for myelinating neurons in the brain. The generation of oligodendrocytes is crucial for recovery from neurodegeneration, as defects in myelination contribute to neuropathologies, including AD [335]. Mitophagy is not only essential for oligodendrocyte generation but also regulates mitochondrial dynamics during oligodendrocyte maturation [336, 337]. In a cuprizone (CPZ)-induced demyelination model, elevated FKBP5 activates the PINK1-Parkin mitophagy pathway, creating a favorable environment for myelin regeneration by counteracting the PPAR-γ-mediated mitochondrial survival mechanism [338]. Mitophagy is also crucial for maintaining the function and survival of OPCs during both development and repair. In summary, mitophagy in both neuronal and non-neuronal cells is a fundamental process that preserves mitochondrial homeostasis and function in a healthy brain. Impairments in mitophagy may contribute to neuronal aging and the pathogenesis of NDDs [329].

### Mitochondrial dysfunction and mitophagy in AD

#### Mitochondrial dysfunction in AD

AD is characterized by progressive cognitive decline, primarily driven by the accumulation of Aβ in senile plaques and hyperphosphorylated tau in neurofibrillary tangles, leading to synaptic dysfunction and neurodegeneration [24]. Mitochondrial dysfunction is among the earliest pathological events in AD [339] and is evident even before significant plaque pathology develops. Energy metabolism deficits in AD include reduced ATP production due to OXPHOS defects and impaired glucose metabolism, contributing to decreased cerebral energy supply [340, 341]. Increased oxidative stress plays a pivotal role in AD pathogenesis, with elevated ROS levels inducing lipid peroxidation, protein oxidation, and mtDNA damage [342]. This oxidative damage is exacerbated by impaired antioxidant defenses, including reduced superoxide dismutase (SOD) and glutathione (GSH) levels [341]. mtDNA is critical for maintaining mitochondrial function, and the accumulation of mtDNA deletions and mutations ultimately compromises mitochondrial integrity [340]. These mutations are associated with pronounced cognitive deficits in AD [340]. A significant increase in mtDNA mutations has been reported in early-stage AD using high-precision next-generation sequencing methodologies, with evidence suggesting that these mtDNA mutations arise primarily from replication errors rather than oxidative damage [343]. Additionally, modifications such as increased methylation in the D-loop region may influence mitochondrial function by regulating transcription [340]. Calcium dysregulation, a crucial feature of AD, impairs mitochondrial calcium buffering, thereby increasing neuronal susceptibility to neurodegeneration by promoting synaptic dysfunction and neuronal death [344]. Rheb (Ras homolog enriched in brain) and Snapin (SNARE-associated protein) are also key regulators of mitochondrial homeostasis at synapses. Rheb facilitates mitophagy by targeting damaged mitochondria for autophagic degradation, while dynein–Snapin-mediated retrograde transport facilitates the clearance of mitophagosomes from synaptic terminals [345].

Mitophagy plays a pivotal role in the pathogenesis of AD by serving as a fundamental quality control mechanism essential for maintaining brain health. It facilitates the removal of damaged mitochondria, mitigates ROS accumulation, attenuates neuroinflammation, and restores mitochondrial homeostasis and function [316, 346, 347]. AD-associated factors Aβ, tau**,** and *APOE* ε4 induce mitochondrial stress, leading to the release of mtDNA, which activates the cyclic GMP–AMP synthase (cGAS)-stimulator of interferon genes (STING) pathway in microglia and neurons, thereby triggering inflammatory responses [348, 349]. Pathological Aβ and tau proteins impair mitophagy, resulting in the accumulation of damaged mitochondria within neurons. This mitochondrial dysfunction exacerbates neuronal stress, synaptic failure, and ultimately, neurodegeneration and cell death in AD [350]. Conversely, stimulation of mitophagy through interventions such as NAD⁺ supplementation, urolithin A, and actinonin has been shown to reverse memory impairment via PINK1-, Parkin-1-, or DCT-1 (DAF-16/FOXO-controlled germline-tumor affecting 1)-dependent pathways. Furthermore, mitophagy activation reduces the accumulation of insoluble Aβ₁–₄₂ and Aβ₁–₄₀ while preventing cognitive decline in APP/PS1 mouse models by promoting microglial phagocytosis of extracellular Aβ plaques and suppressing neuroinflammation. Additionally, mitophagy enhancement has been demonstrated to abolish tau hyperphosphorylation in human neuronal cells and to restore memory function in transgenic tau nematodes and mice [351]. Collectively, mitophagy plays a neuroprotective role by improving Aβ and tau pathology and reversing AD-associated cognitive deficits.

Synaptic plasticity and neurotransmission activity are highly dependent on functional mitochondria to fulfill the energy demands required for neuronal communication. Impaired mitophagy disrupts this delicate balance, leading to synaptic dysfunction and cognitive decline in AD models by exacerbating Aβ and Tau accumulation through increases in oxidative damage and cellular energy deficits [347]. This impairment in MQC not only drives the progression of AD but also suggests a bidirectional relationship where mitochondrial dysfunction can act both upstream and downstream of Aβ and tau pathology. This interplay creates a self-perpetuating cycle: mitochondrial dysfunction leads to synaptic failure and neurodegeneration, which in turn exacerbates mitochondrial impairment, further promoting Aβ and tau accumulation. This vicious cycle accelerates cognitive deficits and disease progression, highlighting mitophagy as a crucial regulatory mechanism in AD pathogenesis, ultimately leading to cognitive deficits [347, 352].

#### Mitophagy in AD

Basal mitophagy levels are reduced by approximately 30–50% in post-mortem hippocampal brain samples from AD patients compared to sex- and age-matched cognitively normal individuals [351]. The mechanisms underlying AD-associated mitophagy defects likely involve disruptions in multiple signaling pathways. Notably, dysregulated expression of key mitophagy regulators, such as PINK1 and Parkin, has been identified in AD brain biopsies, leading to impaired mitophagy via the PINK1/Parkin-mediated ubiquitin–proteasome pathway [347, 352]. Additionally, mitophagy dysfunction in AD may stem from defective autophagosome formation and insufficient PARK2 signaling [353], The accumulation of aged and dysfunctional mitochondria in AD appears to be further linked with the downregulation of multiple genes critical for autophagy and mitophagy. These include OPTN, Autophagy-related genes (ATG5, ATG12), Beclin-1 (Bcl-1), Phosphoinositide 3-kinase class III (PI3K class III), Unc-51-like kinase 1 (ULK1), AMBRA1, BNIP3, BNIP3L, FUN14 domain-containing protein 1 (FUNDC1), Voltage-dependent anion-selective channel 1 (VDAC1), and Valosin-containing protein (VCP/P97) [352, 354]. Defective mitophagy in AD may result from impairments at multiple stages of the process, including mitophagosome formation and fusion with lysosomes. This is exemplified by reduced recruitment of microtubule-associated protein 1 light chain 3 (LC3) to mitochondria, a critical step for mitophagosome biogenesis [351, 352]. Furthermore, dysfunction in AMPK signaling exacerbates mitophagy defects by impairing mitochondrial clearance [351, 352]. Defective mitophagy in human sporadic Alzheimer’s disease (SAD) brains (Braak stage IV-VI) was accompanied by an increase of the LC3-II/I ratio, p62 accumulation**,** and a reduction in PINK1 and Parkin levels in the mitochondria-enriched fraction [352, 355]. Moreover, APP C-terminal fragments (APP-CTFs) accumulation specifically drives mitochondrial dysfunctions and mitophagy failure in AD pathology [355].

The AD prefrontal cortices also exhibit reduced levels of Disrupted in Schizophrenia 1 (DISC1), a critical regulator of axonal mitochondrial transport and mitophagy. DISC1 functions as a mitophagy receptor by binding LC3-II via its LC3-interacting region (LIR) motif, and its depletion may contribute to impaired mitochondrial clearance [356]. The entorhinal cortex (EC) demonstrated mitochondrial dysfunction and mitophagy impairment as early as AD stages I–II, even in the absence of detectable alterations in the frontal cortex area 8 (FC). This suggests that the interaction between mitochondrial dysfunction, defective mitophagy, Aβ, and pTau accumulation may trigger the pathogenesis of AD during its prodromal stages [339, 352, 357]. Several key factors in AD, including tau, Aβ, APP-CTFs, APOE4, and PS1 mutants, impair mitophagy to varying extents. Therapeutic approaches aimed at rectifying these pathological proteins have been shown to significantly mitigate some hallmark features of AD. These findings place mitophagy dysfunction as a pivotal mechanism contributing to mitochondrial pathology in AD. Addressing mitophagy impairments through targeted therapeutic strategies may offer novel avenues for mitigating mitochondrial dysfunction and its downstream effects on neurodegeneration in AD [352].

### Mitochondrial dysfunction and mitophagy in PD

#### Mitochondrial dysfunction in PD

PD is a late-onset neurodegenerative disorder characterized by the progressive degeneration of dopaminergic neurons (DANs) in the substantia nigra pars compacta (SNc), resulting in both motor and non-motor symptoms [358]. It is a complex, age-related, multifactorial disorder influenced by both genetic predisposition and environmental factors [359]. Among the various pathological mechanisms involved in PD, mitochondrial dysfunction is recognized as a central characteristic and a key driver of disease progression [358–360].

Mitochondrial dysfunction has been implicated in the neurodegenerative processes of PD for over three decades. The identification of familial PD-associated genes, PINK1 and parkin, and their roles in regulating mitophagy have further emphasized the critical involvement of MQC in PD pathogenesis [358–361]. In PD, deficits in dopamine release from SNc occur before the overt neurodegeneration of DANs. Additionally, several key cellular changes precede DAN degeneration, including impairments in intracellular trafficking, calcium homeostasis, mitochondrial function, and mitophagy [358, 359]. Dopamine release deficits in PD may arise from dysfunctions in synaptic vesicle (SV) exocytosis, trafficking, loading, endocytosis, recycling, synaptic protein accumulation, SV pool damage, and non-cell autonomous impairments [358]- all of which may be linked to mitochondrial dysfunctions as mitochondria play a crucial role in stabilizing cellular energetics, calcium, and metabolic homeostasis.

Mitochondrial dysfunction in the SNc cell bodies is prominent in PD. Increased mtDNA deletions have been specifically found in postmortem SNc of PD patients, which are associated with ETC deficiency and selective neuronal loss in PD [362]. Higher levels of somatic mtDNA deletions in PD are not accompanied by an increased mtDNA copy number, suggesting impaired mtDNA homeostasis. This contrasts with aging individuals, who exhibit mtDNA depletion and a typically elevated mtDNA copy number as a compensatory response [363]. In PD, an increased SNCA copy number in nigral dopaminergic neurons leads to pathological α-syn accumulation, resulting in mitochondrial protein import inhibition, mitochondrial membrane depolarization, and impaired cellular respiration [359]. Respiratory complex I (CI) deficiency is consistently observed in SNpc dopaminergic neurons in idiopathic PD (iPD). While the causes of this deficiency remain unclear, CI inhibition or mutations in mtDNA regulatory gene are known to induce dopaminergic neurodegeneration [364–367]. iPD exhibits molecular and motor heterogeneity related to CI deficiency, which may affect the dopaminergic substantia nigra to varying degrees [367]. CI deficiency disrupts ETC, reducing ATP production, increasing oxidative stress and intracellular calcium, impairing mitochondrial protein import, and influencing apoptosis, neuroinflammation, α-syn aggregation, and ER stress—all of which are key contributors to neurodegeneration [368].

Excessive ROS production in PD neurons and glial cells primarily originates from multiple sources, including mitochondrial dysfunction, neuroinflammatory cells, and dopamine metabolism. Additionally, other pathways contribute to ROS generation, involving iron, calcium, neuromelanin, glutathione, and lipid metabolism. Alterations in key PD-associated proteins, including α-syn, leucine-rich repeat kinase 2 (LRRK2), PINK1, parkin, DJ-1, F-box only protein 7 (FBXO7), and ATPase Cation Transporting 13A2 (ATP13A2), can disrupt mitochondrial function, leading to increased ROS production and heightened susceptibility to oxidative stress [369, 370]. ROS production and its effects interact with mitochondrial dysfunction through a vicious cycle, wherein ROS-induced damage to mtDNA and other mitochondrial components progressively impairs mitochondrial-ROS physiological function. This disruption ultimately compromises survival-dependent cellular homeostasis in neurons and glial cells, contributing to neurodegenerative pathology.

In midbrain dopaminergic (mDA) neurons derived from heterozygous GBA1-PD human induced pluripotent stem cells (hiPSCs), mitochondrial Ca ^2^⁺ release was already reduced at the earliest developmental stages analyzed. This reduction correlated with impaired mitochondrial function and ATP production, and downregulation of cytosolic phospholipase A2 (PLA2G6), another familial Parkinson's disease (fPD)-associated protein. At later stages, these alterations led to a decline in spontaneous synaptic activity in mature GBA1-PD mDA neurons [371, 372]. Both α-syn oligomerization and dysregulated Ca^2^⁺ homeostasis have been identified as early pathological phenotypes in induced pluripotent stem cell (iPSC)- mDA precursors, as well as neurons from fPD cases with SNCA (encoding α-syn protein) mutations [371, 373]. In SNCA-mutant mDA neurons, α-syn aggregation and dysregulated Ca^2^⁺ homeostasis were followed by"late"pathological phenotypes with many side effects. These include mitochondrial and lysosomal dysfunction, increased oxidative stress, enhanced autophagic clearance of dysfunctional mitochondria, impaired excitability, and reduced viability [371, 373].

A growing body of evidence strongly suggests that dysregulated Ca^2^⁺ homeostasis may be one of the earliest pathological events in neurons predisposed to PD. Beyond its critical role in neuronal physiology, Ca^2^⁺ acts as an essential second messenger involved in various cellular processes, including mitochondrial energy production, lysosomal function, regulation of gene expression and protein activity, and apoptosis. Notably, these processes are frequently disrupted in PD and other neurodegenerative disorders [371]. Collectively, the unique reliance on Ca^2^⁺ signaling and the inherently low Ca^2^⁺ buffering capacity of DANs in SNc [371], combined with their intrinsic characteristics, contribute to their selective vulnerability in PD pathogenesis [374]. Some of these characteristics include a high density of L-type Ca^2^⁺ channels (LTCCs), susceptibility to α-syn aggregation, and the presence of mutant PD-related genes (e.g., LRRK2, DJ-1, Parkin, and PINK1). Additionally, the high mitochondrial energy demand and increased oxidative stress burden further exacerbate their susceptibility to the loss and degeneration of DAN in SNc [371].

In addition to its presence in the cytosol and nucleus of neurons, α-Syn has been shown to localize to mitochondria [375]. A-syn has also been implicated in the disruption of normal mitochondrial functions, including dysregulated fission/fusion, increased ROS generation, impaired Ca^2+^ uptake, and reduced ATP production [376–378]. Excessive α-Syn accumulation at endoplasmic reticulum–mitochondria (ER-mito) contact sites, along with its aggregation or enhanced interactions with other mitochondria-associated ER membrane (MAM) proteins, may contribute to pathological alterations in mitochondrial function and morphology [377]. Meanwhile, mitochondrial dysfunction exacerbates oxidative stress, further promoting α-syn misfolding and aggregation in a vicious cycle [379]. Impaired mitophagy leads to the accumulation of dysfunctional mitochondria, contributing to intracellular neuronal toxicity in PD. Additionally, damaged mitochondria release a wide array of danger signals such as mtDNA, ROS, and cytochrome c, which activate neuroinflammation and microglial activation, further exacerbating PD pathology [380–382].

#### Mitophagy in PD

In PD, several key impairments in the mitophagy mechanism have been identified. In the PINK1/Parkin pathway, PINK1 accumulates on damaged mitochondria and recruits Parkin to promote mitophagy [383]. Mutations in PINK1 or Parkin are commonly found in familial PD that disrupt this process [383]. Defects in Parkin-mediated ubiquitination of mitochondrial outer membrane proteins also prevent the proper recruitment of autophagy receptors such as p62, NDP52, and OPTN, leading to impaired mitophagy [384]. Additionally, oxidative stress and environmental toxins can impair the PINK1/Parkin pathway [383]. Mutations in key proteins, including α-Syn, LRRK2, protein DJ-1 (DJ-1), F-box protein 7 (Fbxo7), and vacuolar protein sorting 35 (VPS35), have been shown to further affect PD pathogenesis through pathways related to Parkin-mediated mitophagy [89]. Moreover, dysfunctions in BNIP3/NIX- and FUNDC1-mediated mitophagy pathways have also been implicated in PD pathology [385, 386]. In PD, lysosomal dysfunction caused by GBA1 mutations and α-Syn accumulation can impair the completion of mitophagy [387]. Furthermore, an imbalance in mitochondrial fission and fusion, due to altered expression of Drp1 (a fission protein) and Mfn1/2 (fusion proteins), hinders efficient mitophagy [388], further exacerbating mitochondrial dysfunction.

### Mitochondrial dysfunction and mitophagy in HD and ALS

#### Mitochondrial dysfunction and mitophagy in HD

HD is a monogenic disorder caused by the expansion of cytosine–adenine–guanine (CAG) trinucleotide repeats in the *HTT* gene, leading to mHTT–associated cellular toxicity, particularly in the striatum of the brain [389]. Mitochondrial dysfunction is thought to play a central role in neuronal dysfunction and cell death of HD pathogenesis [389]. The key mitochondrial impairments characteristic of HD include altered mitochondrial membrane potential, impaired respiratory chain enzyme activity and energy production, and increased ROS generation. HD mitochondria can also demonstrate disrupted Ca^2^⁺ homeostasis and buffering capacity, imbalances in mitochondrial fission and fusion, mtDNA damage, defective mitophagy, and mitochondrial ferroptosis [390]. While the precise mechanism underlying these mitochondrial dysfunctions remains unclear, mHTT appears to play a crucial role in their development. mHTT has been shown to reduce mitochondrial membrane potential and induce mitochondrial fragmentation [391]. Additionally, mHTT suppresses mitochondrial biogenesis and energy metabolism by transcriptionally repressing PGC-1α (peroxisome proliferator-activated receptor gamma coactivator-1 α) in HD knock-in mice [392], further exacerbating mitochondrial impairment.

mHTT impairs mitophagy through multiple potential mechanisms. mHTT reduces *PINK1* expression and prevents its accumulation on depolarized mitochondria, thereby blocking Parkin recruitment [393–395]. However, one study suggests that while mHTT does not affect Parkin-mediated mitochondrial ubiquitination, it reduces the targeting of mitochondria to autophagosomes [393]. Additionally, mHTT aggregates can sequester mitophagy machinery by interfering with autophagy receptors such as OPTN, NDP52, and p62 [394, 396, 397]. Furthermore, mHTT hampers the fusion of autophagosomes with lysosomes, disrupts lysosomal acidification [398], and impairs subsequent protease activity, further compromising mitochondrial turnover. mHTT also contributes to mitochondrial fission–fusion dysregulation by promoting excessive Drp1 activation while downregulating MFN1/2 and OPA1 expression [399–401]. Moreover, mHTT disrupts TFEB (Transcription Factor EB) nuclear translocation, leading to reduced expression of autophagy and lysosomal genes, thereby exacerbating mitophagy impairment [402].

#### Mitochondrial dysfunction and mitophagy in ALS

ALS is a fatal neurological disorder characterized by the progressive loss of upper and lower motor neurons. Increasing evidence suggests that mitochondrial dysfunction plays a key role in ALS pathogenesis [403]. Mitochondrial impairments occur at multiple levels during the early stages of ALS, with morphologically abnormal mitochondria-characterized by swelling, vacuolation, fragmentation, or aggregation-observed in both ALS animal models and patients [403]. ALS-associated mutations in Zn/Cu Superoxide Dismutase 1 (SOD1), TDP-43, Fused in Sarcoma (FUS), and the chromosome 9 opening reading frame 72 (C9orf72) disrupt mitochondrial OXPHOS [403], leading to defects in ETC complexes I and IV, further impairing ATP production [403]. This mitochondrial bioenergetic failure is particularly detrimental to motor neurons, which have high energy demands [403]. Mutant SOD1 aggregates on mitochondria contribute to excessive ROS production in ALS [404]. Additionally, ALS-linked TDP-43 and FUS delocalization exacerbates mitochondrial oxidative damage, including mtDNA damage [405]. Since TDP-43 is a primary component of cytoplasmic aggregates in postmortem ALS tissue, these aggregates are recognized as a hallmark of 97% of ALS cases, with mutant TDP-43 emerging as a causative factor in ALS [405]. Although TDP-43 is involved in multiple pathological processes in ALS, growing evidence highlights its particularly detrimental impact on mitochondria.

TDP-43 aggregates disrupt mitochondrial architecture, ER-mitochondria contacts, mitochondrial dynamics, and axonal transport. They also impair mtDNA transcription, mitophagy-related gene expression, ATP generation, calcium homeostasis, and overall metabolic balance [403, 406–409], further exacerbating neuronal degeneration.

Mitophagy impairments play one of the most significant roles in ALS pathogenesis. Several ALS-linked genes encode core autophagy proteins, such as p62/SQSTM1, OPTN, and TBK1, while others influence autophagy function, including C9orf72, FUS, TDP-43, VAPB (Vesicle-Associated Membrane Protein-Associated Protein B), UBQLN2 (Ubiquilin-2), VCP, CHMP2B (Charged Multivesicular Body Protein 2B), ALS2 (Alsin), FIG4 (FIG4 Phosphoinositide 5-Phosphatase), TUBA4A (Tubulin Alpha 4 A), PFN1 (Profilin-1), and DCTN (Dynactin Subunit 1). Alterations in these proteins may contribute directly to mitophagy defects in ALS [410–412]. Mitochondria-associated TDP-43 has been shown to interfere with Parkin-mediated mitophagy through its interaction with PHB2 and VDAC1 [413]. Additionally, Parkin can ubiquitinate TDP-43, and its overexpression has been demonstrated to mitigate TDP-43-induced cellular toxicity [414]. Furthermore, TDP-43 disrupts mitophagy by directly regulating Parkin and PINK1 [415, 416], exacerbating mitochondrial dysfunction to a greater extent in ALS.

**Fig. 1:**
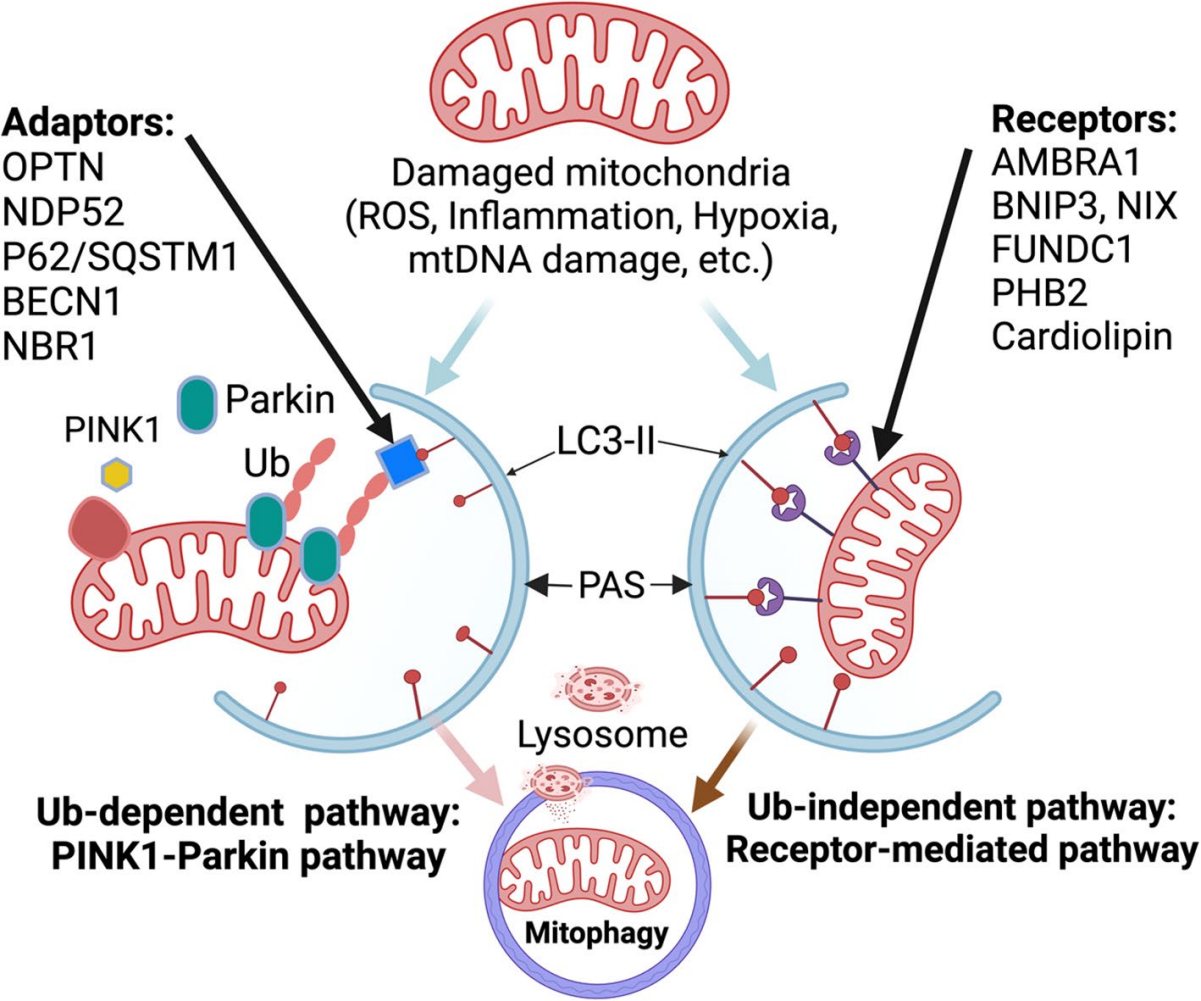
Ub-Dependent and Ub-Independent Mitophagy. Damaged mitochondria can result from various factors, including mtDNA damage, ROS, hypoxia, inflammation, pathogenic proteins, and both genetic and environmental insults, which are commonly associated with the progression of NDDs and cancer. These damaged mitochondria can be removed through Ub-dependent and Ub-independent mitophagy mechanisms. In the Ub-dependent pathway, mitochondrial outer membrane proteins (e.g., MFN1/2, VDAC1, TOM20) are ubiquitinated by Parkin, which is activated by PINK1. This tagging of proteins leads to the recruitment of Ub-binding mitophagy adaptors (e.g., OPTN, NDP52, P62/SQSTM1, BECN1, NBR1), which then interact with LC3 on autophagosomes, facilitating the engulfment and degradation of the damaged mitochondria. The Ub-independent pathway involves mitophagy receptors (e.g., AMBRA1, BNIP3/NIX, FUNDC1, PHB2, cardiolipin), which are directly activated by mitochondrial damage and bind to LC3, promoting mitochondrial degradation

In SOD1^G93A^ ALS mice, significant increases in autophagosomes and degenerated mitochondria are observed in the neuromuscular junction (NMJ) presynaptic terminals. This is accompanied by the downregulation of key mitophagy regulators, including p62/SQSTM1, BNIP3, PINK1, and Parkin, in motor neurons [417]. Recent studies indicate that overactivation of the ERK1/2 (extracellular signal-regulated kinase 1/2) pathway in SOD1^G93A^ ALS mice induces TSPO overexpression, which inhibits mitophagy as a result [418]. Additionally, mutations in proteins such as OPTN and TBK1 impair the recruitment of mitophagy receptors and reduce their interaction with LC3, leading to inefficient mitophagy and the accumulation of damaged mitochondria [411]. Lysosomal dysfunction further exacerbates these mitophagy impairments [419], compounding mitochondrial dysfunction in ALS.

Disturbance of mitochondrial networks and mitophagy dysfunction are key pathological features shared across various NDDs, including AD, PD, HD**,** and ALS. These disorders exhibit mitochondrial fragmentation, bioenergetic and metabolic failure, and defective mitophagy, all of which contribute to neuronal dysfunction and degeneration. Mitochondrial impairment disrupts energy homeostasis, increases oxidative stress, and triggers neuroinflammation, exacerbating disease progression. Understanding these shared mechanisms may provide insights into developing targeted therapies aimed at restoring mitochondrial function and enhancing mitophagy across multiple neurodegenerative conditions.

#### Relationship of mitochondrial dysfunction and mitophagy in NDDs

Mitochondrial dysfunction refers to critical impairments in the structure, function, or regulation of mitochondria that compromise their ability to support cellular homeostasis, particularly in energy metabolism, redox balance, calcium buffering, and programmed cell death. Mitochondrial dysfunction is a pathological trait of all NDDs**,** which is often characterized by impaired oxidative phosphorylation, elevated oxidative stress, dysregulated calcium handling, abnormal dynamics, and defective MQC**,** such as mitophagy. Mitochondrial dysfunction is regarded as the central pathological process in the etiology of NDDs [420–422].

In NDDs, mitochondrial impairments can hinder the mitophagy process, leading to the accumulation of defective mitochondria and neuronal damage. Elevated mtROS levels can damage mitochondrial components and mitophagy-related proteins, disrupting the mitophagy process [383, 423]. Mitochondrial dynamics are closely integrated with mitophagy—imbalances in mitochondrial fission and fusion processes can lead to fragmented mitochondria that are less efficiently cleared by mitophagy, and excessive fission can overwhelm mitophagy capacity [424]. Irreparably damaged mtDNA may trigger mitophagy [425], and accumulation of mtDNA damage can impair mitophagy gene expression and signaling [426].

Insufficient mitophagy leads to the accumulation of dysfunctional mitochondria. This results in intracellular ATP levels decreasing and ROS levels increasing. Excess mitochondrial ROS reacts with proteins involved in the mitophagy process in the development of cancer and NDDs [427]. Conversely, excessive activation of mitophagy can cause cell damage and death [428]. α-syn has been shown to induce neuronal death by promoting mitophagy in A53T mutant mice [89]. Prolonged dysregulation of mitophagy flux is often harmful to neuronal health in neurodegeneration [383].

Mitochondrial dysfunction is a prominent feature in neurons of preclinical AD patients with mild cognitive impairments. Impaired mitophagy has also been observed in the early stages of AD and disrupts a range of biological processes. In AD, a vicious cycle forms among Aβ aggregation/Tau hyperphosphorylation, mitochondrial dysfunction**,** and defective mitophagy, each process exacerbating the others and accelerating disease progression [346, 429, 430]. Similar pathogenic cycles have been reported in other NDDs. In PD, α-Synuclein aggregates and mutations in PINK1/Parkin compromise mitophagy and contribute to neurodegeneration [430–432]. Likewise, mHTT in HD and TDP-43/SOD1 mutations in ALS have been linked to mitochondrial damage and mitophagy dysfunction [430]. Collectively, mitochondrial dysfunction and mitophagy impairment are inseparably linked, forming a pathogenic feedback loop that exacerbates neurodegeneration in NDDs.

## Mitophagy as the master checkpoint in cancer and NDDs

Mitochondria function as metabolic hubs and signaling platforms, playing an essential role in various cellular processes. Mitochondrial dysfunction can severely impact cellular fitness and contribute to disease [433]. This dysfunction is a hallmark of both cancer and NDDs (Table 1). However, the underlying mechanisms and consequences diverge due to the distinct cellular fates of cancer cells, which undergo uncontrolled proliferation, versus neurons, which experience degeneration and cell death. This fundamental distinction is etiologically driven. In cancer, mitochondrial dysfunction arises because of oncogene activation or the loss of tumor suppressors, which results in mitochondrial functions being reprogrammed to support tumor growth and survival [11, 55, 434]. In contrast, in NDDs, mitochondrial dysfunction is primarily triggered by the accumulation of neuropathological proteins, such as Aβ/tau (AD), α-syn (PD), mHTT (HD), and TDP-43 (ALS), leading to progressive neuronal damage and cell death [22, 24, 78].

**Table 1:** Overview of mitochondrial dysfunction in cancer vs. NDDs

Feature	Cancer	NDDs
Mitochondrial Biogenesis	Increased biogenesis to meet high energy and anabolic demands	Impaired biogenesis leading to energy failure
Mitochondrial Metabolism	Metabolic shift towards glycolysis (Warburg effect)	Decreased ATP production due to OXPHOS defects
Mitochondrial Dynamics	Increased fusion (maintains metabolic flexibility)	Imbalance: excessive fission causes mitochondrial fragmentation
Oxidative Stress & ROS	Moderate ROS increase, promotes cancer proliferation and survival	Excessive ROS leads to oxidative stress and neurodegeneration
Calcium Homeostasis	Altered, affecting cell signaling	Dysregulated, affecting neuronal signaling
mtDNA Mutations	Frequent, contributing to cancer progression, but tolerated due to metabolic reprogramming	Accumulation, lead to loss of mitochondrial function, aging and neuronal death
Apoptosis Regulation	Suppressed to allow uncontrolled cell survival	Increased apoptosis contributes to neurodegeneration
Mitophagy Regulation	Inhibited to prevent apoptosis and maintain damaged mitochondria	Overactivated or impaired, leading to neuronal loss

Cells have evolved complex and robust MQC systems to detect mitochondrial dysfunction and mitigate damage, ensuring cell survival and homeostasis [435, 436]. This is primarily achieved through mitophagy, which plays a crucial role in maintaining mitochondrial quality, regulating mitochondrial abundance, and adapting to environmental cues to preserve cellular homeostasis (Fig. 1) [433]. Insufficient mitophagy leads to the accumulation of dysfunctional mitochondria, which can trigger cellular damage [427]. Conversely, excessive mitophagy may result in the depletion of healthy mitochondria, compromising cellular function [437]. Mechanistically, the pathophysiology of NDDs is driven by the progressive accumulation of mitochondrial dysfunction, primarily resulting from mitophagy dysregulation or insufficiency, which leads to neuronal cell death [438]. Cancer initiation and progression are driven by mitochondrial reprogramming and remodeling, primarily through mitophagy adaptation that supports cancer cell survival, metabolic flexibility, and resistance to stress [221] (Table 2).

**Table 2:** Comparison of mitophagy between cancer and NDDs

Feature	Cancer	NDDs
Primary role	Supports cancer cell survival by removing damaged mitochondria, reducing oxidative stress and metabolic reprogramming. Adaptation to stressful conditions like hypoxia and nutrient deprivation. Can also limit tumor growth by restricting energy supply	Maintains neuronal health by removing damaged mitochondria. Impaired mitophagy leads to the accumulation of dysfunctional mitochondrial, contributing to neuronal dysfunction and death
Mitophagy Regulation	Frequently suppressed or hijacked to support cell survival and metabolic adaptation	Can be either impaired or excessive, contributing to disease progression
Main Molecular Players	PINK1/Parkin downregulation prevents excessive mitochondrial clearance, allowing adaptation to stress	PINK1/Parkin pathway is often impaired (e.g., in PD, Parkin mutations prevent mitochondrial clearance)
	BNIP3/NIX-mediated mitophagy is often hijacked to enhance survival under hypoxia	Lysosomal dysfunction due to α-syn, tau, or TDP-43 aggregation inhibits mitophagy completion
	FUNDC1 and AMBRA1 can be co-opted to regulate mitophagy in tumor cells	Excessive mitophagy in ALS or HD leads to excessive mitochondrial loss and bioenergetic failure
	Protein Aggregates impairs mitophagy	Downregulation of multiple genes involved in autophagy and mitophagy in AD (FUNDC1, BNIP3, ATG12)
Mitochondrial Dynamics (Fusion/Fission Balance)	Cancer cells increase mitochondrial fission (via Drp1) to generate small, functional mitochondria that evade mitophagy	Neurons depend on fused mitochondrial networks for high energy demand
	Fusion suppression (via downregulation of Mfn1/Mfn2) prevents mitochondria from being targeted for degradation	Drp1 hyperactivation leads to excessive fragmentation, making mitochondria more susceptible to mitophagy
		Fusion defects prevent mitochondrial functional recovery
Metabolic Impact of Mitophagy	Cancer cells avoid mitophagy-mediated mitochondrial loss, maintaining dysfunctional mitochondria for Warburg effect (aerobic glycolysis)	Impaired mitophagy results in accumulation of defective mitochondria, increasing oxidative stress and neurotoxicity
	Mitophagy suppression prevents excessive energy depletion and apoptosis	Excessive mitophagy depletes mitochondria, leading to synaptic dysfunction and neuronal death
Oxidative Stress Response	Some cancers tolerate high ROS levels, using them for tumor progression	Neurons are highly sensitive to ROS accumulation, leading to protein aggregation (e.g., α-syn, tau, TDP-43, and Aβ)
	Suppressing mitophagy allows ROS signaling to activate oncogenic pathways (e.g., HIF-1α, NF-κB, and p53 suppression)	Impaired mitophagy leads to accumulation of oxidized mitochondrial proteins and lipids
Hypoxia and Mitophagy	Hypoxia-induced mitophagy (via BNIP3/NIX) enhances tumor survival by removing damaged mitochondria	Neurons are sensitive to oxygen fluctuations, and hypoxia can exacerbate mitochondrial dysfunction
	Hypoxia-inducible factors (HIF-1α) regulate mitophagy to allow adaptation	HIF-1α dysregulation contributes to neurodegeneration
DNA Damage and Genomic Instability	Mitophagy suppression prevents excessive mitochondrial turnover, sustaining mtDNA mutations that promote tumorigenesis	mtDNA mutations accumulate in aging and cause mitochondrial dysfunction in NDDs
	Cancer cells use mutant mitochondria to drive metastasis and chemoresistance	Inefficient mitophagy leads to persistent damaged mtDNA, which enhances neurodegeneration
Cell Fate Regulation	Suppressed mitophagy prevents apoptosis by retaining mitochondria with anti-apoptotic signaling	Impaired mitophagy results in cellular senescence and neuronal degeneration
	Cancer cells with dysfunctional mitophagy become resistant to cell death mechanisms (e.g., intrinsic apoptosis via Bcl-2 upregulation)	Loss of mitophagy regulation can trigger excessive neuronal apoptosis (e.g., in AD and PD)
Therapeutic Targeting of Mitophagy	Pro-mitophagy therapies (e.g., autophagy inducers) are being explored to trigger mitochondrial clearance and sensitize tumors to therapy	Restoring mitophagy is a key strategy to remove defective mitochondria in PD, ALS, and AD
	Inhibiting mitophagy can also be a strategy to induce cancer cell death	Inhibiting excessive mitophagy may protect neurons from bioenergetic failure (e.g., in ALS and HD). Inhibiting excessive mitophagy may protect neurons from bioenergetic failure (e.g., in ALS and HD)

**Fig. 2:**
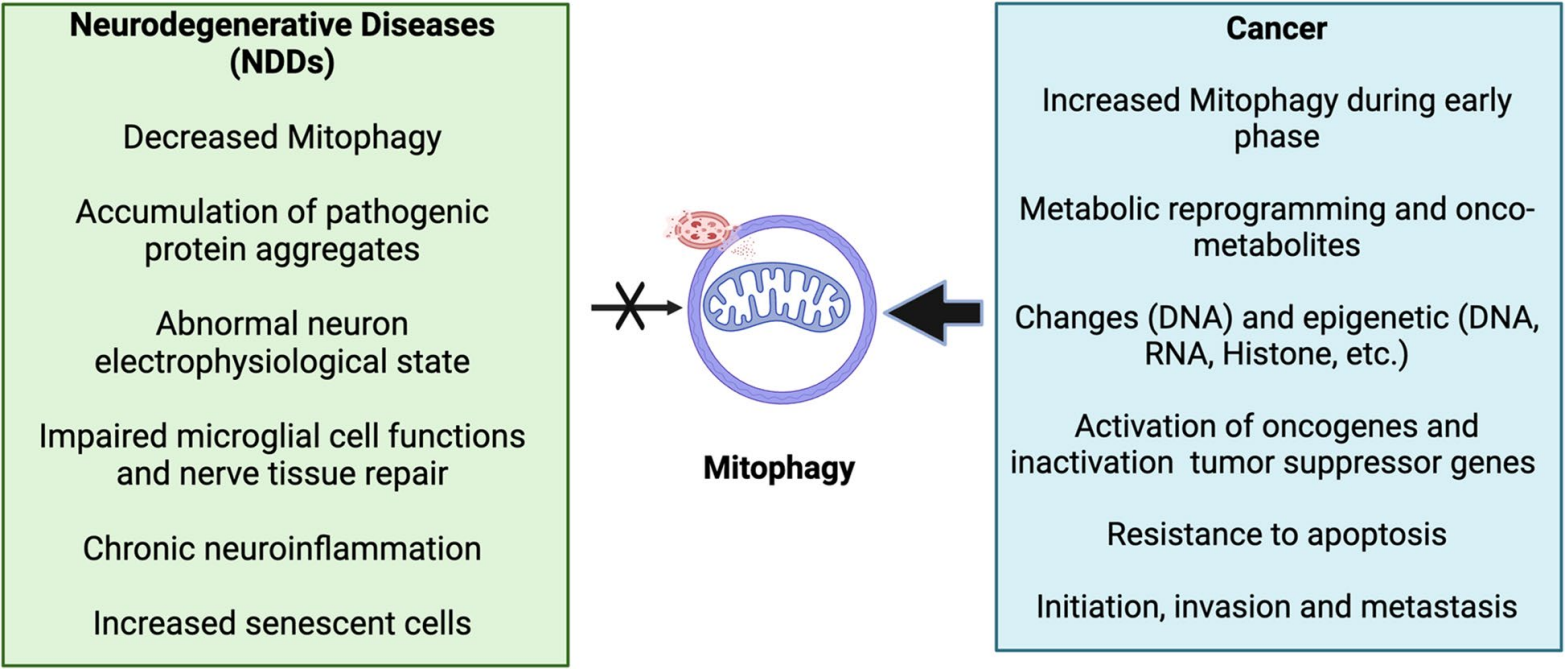
Comparative analysis of mitophagy in NDDs and cancers. Dysregulated mitophagy is a key contributor to mitochondrial dysfunction in both NDDs and cancer, though with distinct pathological consequences. In NDDs, defective mitophagy leads to the accumulation of dysfunctional mitochondria, exacerbating oxidative stress, synaptic and neuronal network dysfunction, neuroinflammation, and ultimately neuronal injury and death. Conversely, during early tumorigenesis, mitophagy serves as a protective mechanism by clearing damaged mitochondria, thereby preventing ROS production, genomic instability, metabolic and proteomic imbalances, and aberrant signaling factors that drive malignant transformation. However, in established malignancies, mitophagy undergoes context-dependent adaptation, either upregulated or suppressed, to support tumor growth, metastasis, and resistance to therapy

The normal functionality of the CNS relies on a steady supply of healthy mitochondria. Mitochondrial dysfunction plays a pivotal role in NDDs such as AD, PD, and HD [352, 439–442]. Impaired mitophagy is a major contributor to mitochondrial dysfunction, leading to the accumulation of damaged mitochondria, disease-related pathogenic proteins and metabolites, senescent cells, and chronic neuroinflammation in the pathogenesis of NDDs [352, 439–442] (Fig. 2). In contrast, carcinogenesis is driven by a complex interplay of factors, including mitophagy-mediated deregulation of oxidative metabolic reprogramming, as seen in the Warburg effect and the accumulation of onco-metabolites. This metabolic reprogramming and these alterations are closely intertwined with genetic and epigenetic changes, which activate oncogenes and inactivate tumor suppressor genes, resulting in stepwise cellular transformation and oncogenesis [99, 443, 444] (Fig. 2).

## Potential therapeutics for NDDs

**Fig. 3:**
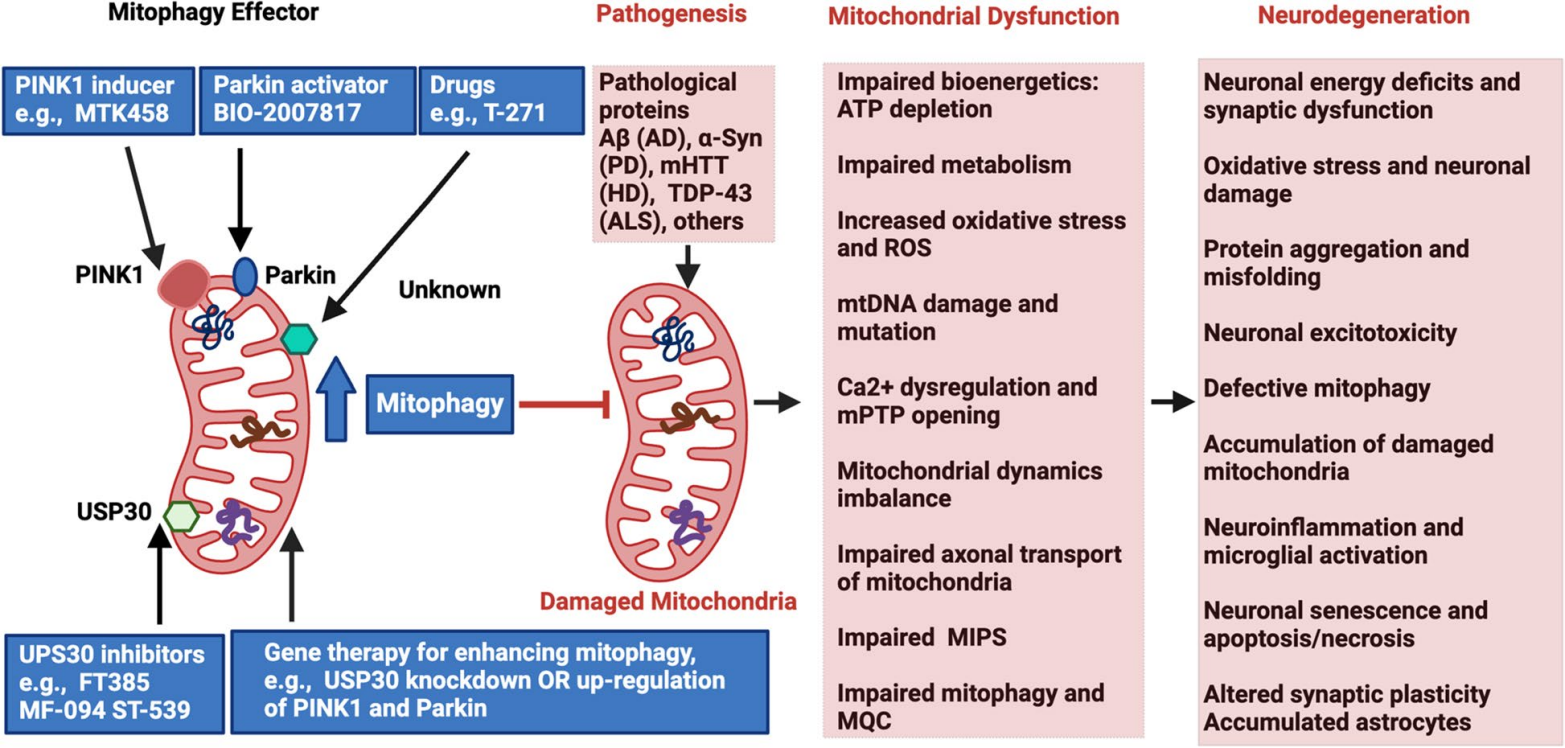
Therapeutic potential of targeting mitophagy in NDDs. NDDs are characterized by progressive neuronal damage and loss, often accompanied by chronic neuroinflammation, reactive gliosis, and the accumulation of pathogenic protein aggregates. Mitochondrial dysfunction, particularly impaired mitophagy, plays a central role in these pathological processes by contributing to oxidative stress, bioenergetic failure, and cellular toxicity. Emerging therapeutic strategies aimed at enhancing mitophagy are being actively explored and hold significant promise for mitigating disease progression and improving clinical outcomes in NDDs

The primary goal for future therapeutic strategies targeting NDDs should be to stimulate mitophagy and promote cell proliferation (Table 3 and Fig. 3). Although done in a malignant way, this is done exceptionally well by tumor cells, meaning that if the concepts utilized by cancer cells can be applied in a non-malignant way to NDD patients, more effective therapies can be created. An example of one such concept is the Warburg effect. The Warburg effect mechanism, commonly observed in cancer cells, consists of a dramatic increase in glucose uptake and lactate production even with normoxic conditions and fully functioning mitochondria. This can be seen in normal growing and proliferating cells, meaning this increase in lactate production is necessary for promoting cell growth, which is needed in NDD therapies. It’s also important to note that lactate is a trigger for mitophagy, which can explain the rapid production of lactate. Cells that are proliferating rapidly need higher mitophagy levels to sustain healthy ROS levels in the newly created cells and to remove dysfunctional mitochondria. Although pushing lactate levels could be a possibility, it is important to explore other possibilities to increase mitophagy levels within NDD patients.

**Table 3:** Common drugs or compounds used as effector of mitophagy

Drugs or Compounds	Mechanism of action	Application
**Mitophagy inducers**		
3,4-Ethyl ester of dihydroxybenzoate	BNIP3-mediated mitophagy	Esophageal cancer
Actinin	Induce PINK1/Parkin pathway mitophagy	AD
AT101 (a novel BH3 mimic)	Induce mitophagy and non-apoptotic cell death	Glioblastoma
Cannabidiol (CBD)	PINK1/Parkin pathway	CML
Ceramide or C18-ceramide	BNIP3-mediated mitophagy	HNSCC, Glioblastoma
Curcumin	Induce mitophagy	Nasopharyngeal cancer
Deferiprone	Induce PINK1/Parkin independent mitophagy	PD
Dihydroergotamine tartrate (DHE)	PINK1/Parkin-mediated mitophagy, Induction of apoptosis	Lung cancer
Dihydromyricetin (DHM)	Induces mitophagy	PD/AD, cancer (liver, ovary)
Epigallocatechin-3-gallate (EGCG)	Activation of Sirt1/AMPK/PGC-1a signaling, induce mitophagy	AD/PD
Embelin	Induces mitophagy and apoptosis	PD, cancer (liver, breast)
Fisetin	PINK1/Parkin pathway	PD
Ginsenoside	PINK1/Parkin pathway	Colon cancer
Ketoconazole	PINK1/Parkin-mediated mitophagy, Induction of apoptosis	Liver cancer
LCL-461 (Ceramide analog)	BNI + C5P3-mediated mitophagy	AML
Metformin	Improve PINK1/Parkin pathway mitophagy	AD/PD/HD/ALS
NAD + precursor	Increase the NAD +/NADH ratio, induce mitophagy	AD
Naringin	PINK1/Parkin pathway	PD
Niclosamide	Induce mitophagy by AMPK activation and PINK1/Parkin pathway	PD
Phenanthroline	Mitophagy induction by mitochondrial fragmentation and dysfunction in a DRP1 dependent manner	Cervical carcinoma
PMI	ROS accumulation, p62-mediated mitophagy	Liver cancer, Neuroblastoma
Quercetin	Induces mitophagy	PD/AD, cancer (prostate, cervical, lung, breast, and colon)
Rapamycin	Induce mitophagy and inhibit mTOR functions	Breast and Renal cancer, AD/PD/HD/ALS
Resveratrol	Inhibits ROS, activates mitophagy and AMPK/SIRT1/PGC-1a signal pathway	PD/AD/Dementia, cancer (liver, pancreas, prostate and colorectal)
Salinomycin	Mitophagy induction by mitochondrial depolarization	Prostate cancer
Sorafenib	PINK1/Parkin-mediated mitophagy, Induction of apoptosis, ETC chain inhibition	Liver cancer, Renal Cancer
Spermidine	Promote mitophagy	AD/PD/HD/ALS
Trehalose	Induce mitophagy	AD/PD/HD/ALS
Urolithin A	Induce PINK1/Parkin pathway mitophagy	AD
Ursolic acid	PINK1/Parkin pathway	Lung cancer
Valinomycin	PINK1/Parkin pathway	Liver cancer
Shikonin	Induces mitophagy	AD/PD/HD, cancers (liver, breast, lung, colon)
Sanguinarine	Induce ROS-dependent mitophagy	HCC
**Mitophagy Inhibitors**		
Fluorizoline	PINK1/Parkin pathway inhibition	Lung cancer, Cervical cancer
Liensinine	Blocks autophagosome-lysosome fusion, DRP1-mediated mitophagy inhibition	Breast cancer
MDIVI-1	DRP1-mediated mitophagy	Liver cancer, Oophoroma
Melatonin	PINK1/Parkin pathway, Apoptosis induction, Downregulation of JNK kinase	Cervical carcinoma
PKI-402	Mitophagy inhibition by potent inhibition of PI3K and mTOR	Liver cancer

When considering existing drugs that promote mitophagy, one primary approach for enhancing mitophagy involves the upregulation of Sirt1 expression, which is notably facilitated by ferulic acid. Ferulic acid is synthesized through the metabolic breakdown of dietary anthocyanins by gut bacteria [445]. Numerous cell-culture and animal studies have demonstrated the anti-inflammatory and antioxidant properties of ferulic acid, which have long been harnessed in Chinese medicine for CVD. More recently, its ability to increase Sirt1 expression has been discovered, with research revealing its effects on various cell types, including neural stem cells, chondrocytes, and hepatocytes [446]. Notably, its ability to enhance mitophagy in neural stem cells proves valuable in counteracting the reduced mitophagy levels associated with elevated ROS content in NDDs. Presently, ferulic acid finds its primary usage in anti-aging skin creams and serums, primarily due to the limited extent of research conducted on this compound. A comprehensive investigation of its capabilities is essential to ensure its safety.

Melatonin, an alternative stimulant of Sirt1**,** which has undergone extensive research and examination, has also exhibited promising results in augmenting mitophagy. Melatonin shares numerous advantages with ferulic acid, but it has garnered significantly more research attention and possesses a deeper understanding. By stimulating the activation of the BMAL1(Brain and Muscle Arnt-Like 1) transcription factor, melatonin effectively enhances the transcription of Sirt1 [447, 448]. Furthermore, melatonin directly promotes the mitophagy pathway by increasing the expression of HSPA1L (Heat Shock Protein Family A (Hsp70) Member 1-Like) [449]. When HSPA1L expression is decreased, parkin, a gene that helps regulate the mitochondrial control pathway, impairs mitophagy [450]. In mitophagy, HSPA1L transfers and tethers parkin to the membranes of dysfunctional mitochondria, effectively causing their removal by lysosomes. Through treating human mesenchymal stem cells (hMSCs) that had high oxidative stress content with melatonin, not only did these hMSCs show augmented resistance against ROS-induced apoptosis**,** but the expression of HSPA1L and parkin was also dramatically increased, leading to an increase in mitophagy as well [451].

Looking past the current drugs, the main alternative to lactate in this situation is Ca^2+^. Ca^2+^ also has the positive effects of lactate, with Ca^2+^ being a trigger for mitophagy that can function as a substitute for lactate when observed in the Warburg effect mechanism. It is important to acknowledge that the mitochondrial parameters that activate mitophagy machinery, including structure, membrane potential, etc., are also important facilitators of the mitochondrial capacity for Ca^2+^. Therefore, it stands to reason that increased intake of Ca^2+^ would activate the same mitochondrial parameters that activate mitophagy. Initially, Ca^2+^ overload in mitochondria is associated with mPTP opening, which leads to large-scale mitochondrial alterations and apoptosis. However, preventing mitochondrial Ca^2+^ loading can preserve mitochondrial membrane potential (Ψm) and morphology, protecting cells from apoptosis. Mitochondrial Ca^2+^ accumulation is facilitated by MCU, which relies on Ψm as a driving force. The accumulation of mitochondrial Ca^2+^ and mPTP opening is linked to oxidative stress and the activity of proteins like PINK1 and the mitochondrial Na ^+^/Ca2^ +^ exchanger. Studies have also indicated that changes in mitochondrial Ca^2+^ content can influence the autophagic pathway, further supporting Ca^2+^ as a key player in mitophagy. The ER and its Ca^2+^ stores are also implicated in mitophagy. Proteins like BCL2 and BCL2L1/Bcl-xL, which regulate ER Ca^2+^ levels and release, have anti-autophagic roles and interact with proteins involved in autophagy and mitophagy. The interaction between the ER and mitochondria is crucial, as disruptions in their coupling can trigger mitophagy. Proteins like PARK2 and RHOT1, which sense mitochondrial Ca^2+^ levels, play roles in the regulation of ER-mitochondria contact sites and mitophagy [452]. Although the precise mechanisms of calcium's involvement in mitophagy are not fully understood, the existing evidence suggests its active participation in triggering and modulating the process.

Having discussed how current and future drugs can target mitophagy more effectively, the potential therapeutic role of stimulating Ca^2^⁺ and promoting cell proliferation emerges as a promising avenue for future research. By drawing inspiration from the concepts utilized by cancer cells, it is possible to utilize non-malignant applications of processes, such as the Warburg effect, to develop more effective therapies for NDD patients. Lactate, a trigger for mitophagy, and Ca^2+^, which maintains similar positive effects, emerge as potential candidates to increase mitophagy levels within NDD patients. While lactate offers a plausible option, the intricate involvement of Ca^2+^ in mitochondrial parameters and its capacity to trigger mitophagy provide a more compelling alternative. Furthermore, the interplay between mitochondrial and ER dynamics, coupled with proteins involved in Ca^2+^ regulation and mitophagy, adds another layer of complexity to the potential therapeutic mechanisms that expand past current drugs that upregulate Sirt1 expression. However, in the short term, testing ferulic acid and melatonin as NDD drugs may have potential results. As our understanding of these intricate pathways and mechanisms continues to advance, further exploration and investigation of lactate, Ca^2+^, and other possibilities hold great promise for the development of future therapies for NDDs.

Although there are currently no approved therapeutic agents that specifically and selectively modulate mitophagy in NDDs, enhancing mitophagy has emerged as a promising strategy to restore mitochondrial quality control and delay neurodegeneration [453]. Numerous candidate compounds with mitophagy-modulating properties are summarized in Table 3 and Table 4. Beyond previously mentioned agents such as Urolithin A, Actinonin, and Melatonin, additional compounds show potential in preclinical models. For example, dimethyl fumarate (DMF) confers neuroprotection in PD models by activating the BNIP3/PINK1/Parkin pathway [454]. Apigenin may modulate chronic immune neuroinflammation and mitophagy via the mTOR/AMPK/ULK1 signaling axis [455]. Furthermore, Metformin and Niclosamide have demonstrated potential as adjunct therapies in NDDs due to their ability to influence mitochondrial homeostasis and stimulate mitophagy-related pathways [456, 457].

**Table 4:** Novel selective mitophagy enhancers

Lead compound	Mechanism	Stage of development	Company	Key references
MTX115325	USP30 inhibitor	Phase 1	Mission Therapeutics	WO2021/249909. Ref [314, 463].
FT385	USP30 inhibitor	Preclinical	Forma Therapeutics	WO2019071073. Ref [314].
CMPD39	USP30 inhibitor	Preclinical	Mitobridge	WO2018213150. Ref [314, 507].
VB-08	USP30 inhibitor	Preclinical	Vincere Biosciences	WO2021050992. Ref [314].
Unknown	USP30 inhibitor	Preclinical	Amgen Inc./Carmot Therapeutics	WO2020036940 Ref [314].
ST-539	USP30 inhibitor	Preclinical	The Ohio State University	Ref [461, 508].
NK036	USP30 inhibitor	In vitro assay	Max Planck Institute of Molecular Physiology,	Ref [509].
MF-094	USP30 inhibitor	In vitro assay	Mitobridge, Inc	Ref [510].
ST-480	USP30 inhibitor	In vitro assay	The Hebrew University	WO2024147139
ABBV-1088	PINK1 activator	Phase I (NCT06414798/NCT06579300)	Mitokinin/AbbVie	WO2021168446A1. Ref [314].
MTK458	PINK1 activator	Preclinical	Mitokinin Inc	Ref [511].
BIO-2007817	Parkin activator	Biogen	Biogen	Ref [314, 512].
VNA-318	Mitophagy inducer (target unknown)	Phase I (NCT06721091)	Vandria	US20220105117A1. Ref [314].
T-271	Mitophagy inducer (target unknown)	Preclinical	N/A	US9989518B2, ref [314, 513].

**Fig. 4:**
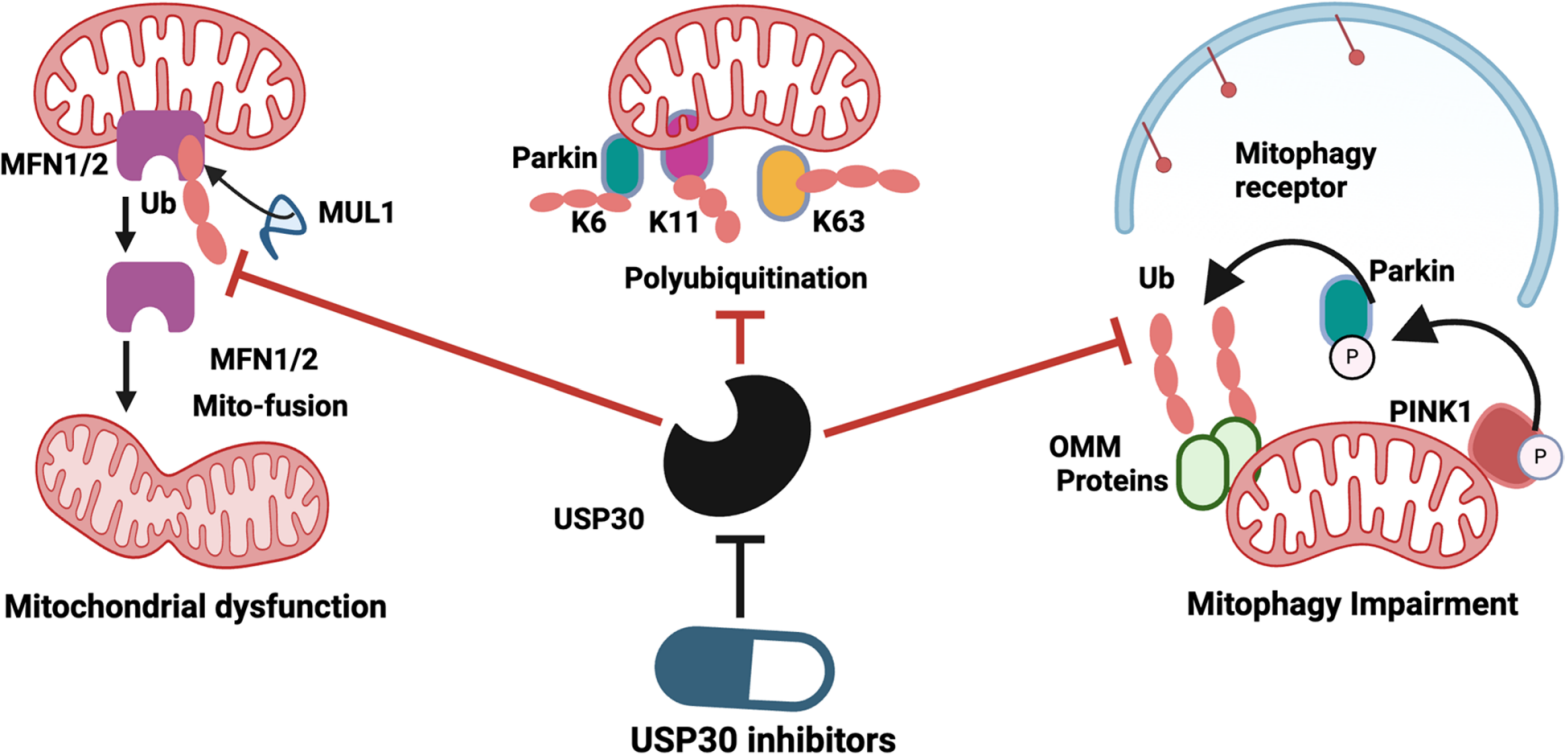
The role of USP30 in Mitophagy. USP30 regulates mitophagy by modulating mitochondrial dynamics and ubiquitination of OMM proteins. Specifically, USP30 deubiquitinates MFN1/2, increasing their stability and promoting mitochondrial fusion. Additionally, USP30 selectively removes both non-canonical (K6- and K11-linked) and canonical (K63-linked) ubiquitin chains from Parkin and key OMM proteins such as TOM20. This process impairs Parkin recruitment, prevents the activation of mitophagy-related OMM proteins, and ultimately suppresses mitophagy, thereby inhibiting the clearance of damaged mitochondria. Pharmacological inhibition of USP30 has been proposed as a therapeutic strategy to enhance mitophagy, facilitating the removal of dysfunctional mitochondria and restoring mitochondrial function

USP30, a mitochondrial deubiquitinase, acts as a negative regulator of mitophagy by counteracting Parkin-mediated ubiquitination of OMM proteins [458]. By removing ubiquitin from key substrates like MFN1/2, VDAC1, and TOM20, USP30 prevents the recruitment of mitophagy adaptors, impairing mitochondrial clearance [459, 460]. Additionally, USP30 influences mitochondrial dynamics by stabilizing mitofusins, promoting fusion over fission, which hinders the isolation and degradation of damaged mitochondria (Table 4) [460]. Inhibiting USP30 (e.g., ST-539) may synergize with AKT/mTOR inhibitors in cancer treatment [461]. USP30 can cause the accumulation of dysfunctional mitochondria in pathological states, and its inhibition has emerged as a promising therapeutic strategy to enhance mitophagy in NDDs and cancer [462] (Fig. 4). MTX115325 protects dopaminergic neurons by promoting mitophagy in preclinical experiments with good oral bioavailability and CNS penetration [463]. The initiation of clinical trials (early 2025) for MTX325 highlights the immense therapeutic potential of targeting USP30 in NDDs such as PD [464]. FT385 (also known as MTX652) is highly selective for USP30, which can repeat the effects of genetic USP30 deletion on mitophagy, and it can also enhance ubiquitylation and degradation of TOM20 without affecting PINK1 protein level [464, 465]. PINK1 activator ABBV-1088 has shown positive outcomes in preclinical models and is now entering phase 1 clinical trials with potential indication for PD (NCT06414798) [314]. ABBV-1088 may be a milestone in mitochondrial-targeted neurotherapeutics as it can directly restore MQC via PINK1 activation. VNA-318, a small-molecule compound, has demonstrated the ability to improve mitochondrial function in preclinical models of Alzheimer’s and Parkinson’s diseases. It has advanced to a Phase 1 clinical trial (NCT06721091), reflecting its translational potential for NDD therapy. These findings underscore the therapeutic relevance of targeting mitophagy and support the need for further translational research to identify and refine mitophagy-specific drugs for clinical use in NDDs.

## Potential therapy for cancer

We’ve established how mitophagy has been associated with detrimental effects in cancer patients. From a biological perspective, it would be assumed that higher mitophagy levels, or cellular removal levels, would prove effective in combating cancer, a disease characterized by rapid cell proliferation. However, the opposite has been demonstrated to be true. High mitophagy levels in cancer patients often increase the therapy resistance of many cancer cells, as well as lower ROS levels. Continuing with this correlation, emerging evidence suggests that future therapeutic strategies should focus on inhibiting mitophagy. In this section, we will explore the intricate interplay between mitophagy, CSC, therapy resistance, and ROS levels, highlighting the potential of modulating mitophagy as a therapeutic strategy to overcome resistance and improve treatment outcomes, as well as possible utilizations of calcium and MCUs to block mitophagy.

In many studies, mitophagy has been implicated in the regulation of CSC subpopulations and the acquisition of chemoresistance [466]. Further, it has been demonstrated that mitophagy plays a role in promoting stemness and chemoresistance in CSCs. In hepatic CSCs, mitophagy suppression inhibits p53 degradation, leading to p53 translocation into the nucleus and the repression of NANOG expression, a critical transcription factor for maintaining stemness [274]. This mechanism reduces the hepatic CSC subpopulation and sensitizes cancer cells to treatment. Similarly, in esophageal squamous cell carcinoma cells undergoing EMT, Parkin-dependent mitophagy increases the expression of the stem cell marker CD44, thereby promoting stemness [467].

Furthermore, mitophagy has been implicated in the metabolic regulation of stemness in cancer cells. For instance, in lung cancer and nasopharyngeal carcinoma, CSCs exhibit reduced mitochondrial mass compared to non-CSCs, suggesting a potential role for mitophagy in maintaining the stem phenotype [468, 469]. Genetic inhibition of mitophagy pathways has shown promise in sensitizing cancer cells to anticancer treatments. Downregulation of PINK1/Parkin- or Rab9a-mediated mitophagy has been linked to increased radiosensitivity and chemosensitivity in cancer cells [470, 471]. Additionally, key mitophagy receptors, such as PINK1, FUNDC1, or AMBRA1, when downregulated genetically, have been shown to sensitize cancer cells to chemotherapy [472, 473].

Therapy-resistant cells, particularly CSCs, often display higher levels of mitophagy, contributing to their resistance to anticancer treatments. Enhanced mitophagy has been implicated in resistance to cisplatin, etoposide, oxaliplatin, and doxorubicin in various cancer cell types. However, impairment of mitophagy has been shown to resensitize drug-resistant cancer cells to treatment [474]. In recent studies, blocking mitophagy using nanomedicine in chemotherapy-resistant ovarian cancer cells restored their sensitivity to treatment, both in xenograft mice and in therapy-resistant cancer patients [475]. The role of mitophagy in CSC chemoresistance is complex, as it may contribute to a higher OXPHOS state within the CSC population to facilitate proliferation [476]. Additionally, by regulating ROS production and mitochondrial transmembrane potential, mitophagy reduces apoptotic cell death, thereby promoting therapy resistance [477]. Overall, targeting mitophagy to limit its activity and increase ROS levels represents a potential therapeutic strategy to sensitize cancer cells, particularly CSCs, to anticancer treatments. By preserving mitochondrial fitness and disrupting the adaptive mechanisms of cancer cells, the inhibition of mitophagy holds promise for overcoming therapy resistance and improving treatment outcomes.

Mitophagy’s role as a cancer inhibitor also lies in its ability to regulate ROS levels. Cancer thrives on elevated levels of ROS by leveraging these moderate increases in oxidation. This is due to the increased antioxidant systems developed by cancer cells. This antioxidant system in the formation of lactate by cancer cells occurs through increased glycolysis, also known as the Warburg effect. The lactate triggers mitophagy to ensure the ROS levels are balanced to benefit cancer cells. However, this antioxidant system also renders cancer cells more sensitive to external factors that increase ROS levels past their limit [478, 479]. A key example of one of these external factors is the limitation of mitophagy, which effectively overwhelms the redox adaptation of cancer cells by increasing ROS levels past the limit for cellular life.

With the evidence supporting these benefits to lowered mitophagy levels (Table 3), it raises the question of how to inhibit mitophagy levels. There are two major drugs that are being explored for mitophagy blocking: Liensinine and Mdivi-1. Liensinine is a major isoquinoline alkaloid that also can inhibit mitophagy through blocking autophagosome-lysosome fusion and prohibiting the excessive accumulation of autophagosomes [480]. When mitochondrial fission occurs, the dysfunctional portion of the mitochondria is taken away by autolysosomes, which are fused autophagosome and lysosomes. When liensinine inhibits this fusion, there aren’t any autolysosomes to remove the dysfunctional portion after mitochondrial fission. There are some issues to liensinine usage, however, research found that inhibition of autophagy/mitophagy by liensinine enhanced doxorubicin-mediated apoptosis by triggering mitochondrial fission, which resulted from dephosphorylation and mitochondrial translocation of DNM1L. By inhibiting mitophagy, there was enhanced cell death, which mirrors the progression of NDDs. Additionally, all previous studies have been conducted on breast cancer patients, and further research must be done regarding its effectiveness in lowering mitophagy levels in other varieties of cancer patients.

These issues bring us to the other prospective treatment Mdivi-1. Although also lacking in clinical research and understanding, the recently discovered compound may be a better prospect for inhibiting mitophagy. This is because of its approach in inhibiting mitophagy. While there are few direct inhibitors of mitophagy, rather than blocking the formation of autophagosomes like liensinine, Mdivi-1 directly inhibits mitochondrial division, the process that causes mitophagy [481]. Mdivi-1 does this by lowering DRP1 levels**,** however, the metabolic mechanism beneath this DRP1 inhibition is still unclear. Studies have shown Mdivi-1 treatment and DRP1 deficiencies to induce mitochondrial fusion, subsequently lowering the necessity for mitophagy-related lysosome activity. Mdivi-1’s applications towards cancer mainly rely on its ability to decrease the DRP1 expression in cancer cells. Studies showed that high DRP1 expression induced fragmented mitochondria in several types of cancer [482]. These fragmented mitochondria have less active OXPHOS than the tubular mitochondria, and this leads to elevated glycolysis levels in cancer cells [483]. By using Mdivi-1 to inhibit mitochondrial fission, it will not only lower mitophagy levels to maintain ROS overload in tumors, but also increase OXPHOS levels and decrease glycolysis levels in patients. Similarly to the issues found in liensinine, these studies have only been done on rat models of acute lung injury (ALI) and lung cancer, requiring further study and understanding of their translatability in humans.

Based on the issues found in the previous drugs and current compiled literature evidence, it can be said that the answer to inhibiting mitophagy lies in the MCU, which is a protein that facilitates the travel of Ca^2^⁺ from the cell’s ER into the mitochondria [484]. Ca^2^⁺ is a trigger for mitophagy, meaning the utilization of MCU inhibitors can regulate Ca^2^⁺ levels in mitochondria and subsequently lower mitophagy levels in the affected cells [485]. The issue of finding an effective MCU inhibitor is that the most cutting-edge inhibitor, Ru360, has availability and injection flaws [486]. In the future, when a potent MCU inhibitor is discovered, potential cancer therapeutics will have to utilize it to lower mitophagy levels. This will be necessary to target CSC cells, limit therapy resistance, and maintain high ROS levels.

**Fig. 5:**
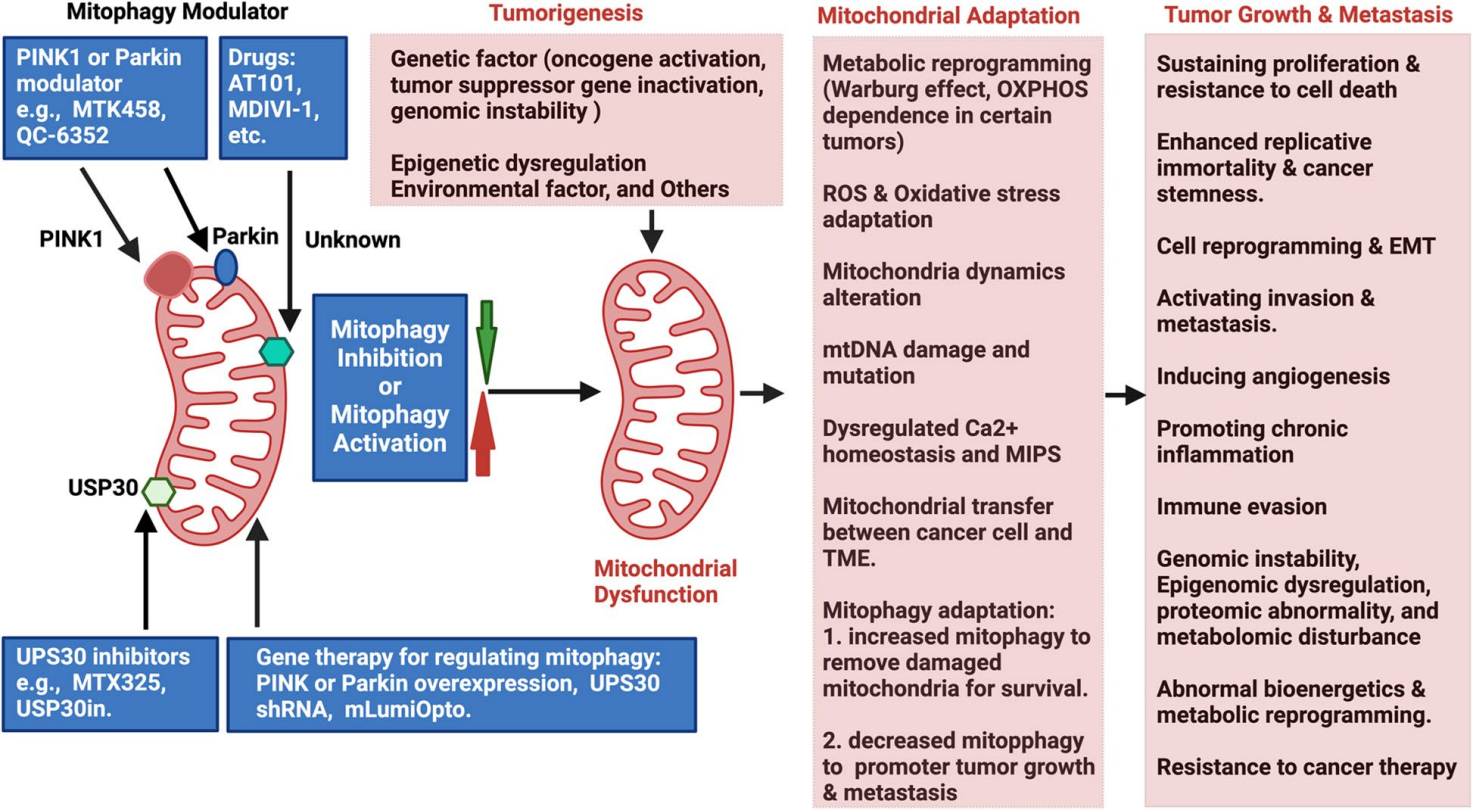
Therapeutic potential of targeting mitophagy in cancer. Tumorigenesis is a highly complex pathological process driven by genetic and environmental factors, including oncogenic activation, inactivation of tumor suppressor genes, DNA damage, and gene dysregulation. These events frequently lead to mitochondrial dysfunction, resulting in the accumulation of damaged mitochondria and the activation of adaptive mitochondrial responses. A critical component of this adaptation is context-dependent mitophagy, which may be either upregulated or suppressed, ultimately contributing to cancer cell survival, proliferation, metastasis, and resistance to therapy—hallmarks of cancer progression. Given the pivotal role of mitophagy in tumor biology, selective mitophagy-targeting therapeutic strategies are being actively investigated and hold significant promise for improving cancer treatment outcomes

In most cancers, mitophagy is upregulated to maintain MQC and energy production, thereby supporting the metabolic demands of rapidly proliferating cancer cells. However, in certain cancers, suppressed mitophagy may contribute to tumor progression through distinct mechanisms. In these cases, a mitophagy inducer could potentially trigger cancer cell death through excessive clearance of mitochondria [487, 488]. The role of mitophagy in cancer initiation and progression is highly intricate and remains insufficiently elucidated. Notably, the dual role of mitophagy observed in some cancer types indicates that both the activation and inhibition of mitophagy could serve as promising strategies for developing anticancer therapies [38] (Fig. 5).

Beyond previously mentioned agents such as Liensinine and Mdivi-1, several other small molecules exert anti-tumor effects through mitochondria-mediated mechanisms, including the modulation of mitophagy. Melatonin has been shown to downregulate the c-Jun N-terminal kinase (JNK) pathway, subsequently reducing Parkin expression and enhancing apoptosis in cervical cancer cells [489]. Fluorizoline, a prohibitin-binding compound, inhibits mitophagy and induces apoptosis in a variety of cancer types [490]. Conversely, mitophagy inducers have also demonstrated anti-tumor efficacy. Ketoconazole activates the PINK1–Parkin mitophagy pathway by downregulating COX-2 expression, thereby promoting apoptosis in hepatocellular carcinoma HCC [489]. Fisetin is a natural flavonoid with antioxidant, neuroprotective, and anti-cancer properties, and it has shown potential in modulating mitophagy-related pathways in tumor suppression [426, 491, 492]. Sorafenib, a multi-kinase inhibitor approved for advanced liver and renal cancers, also demonstrates activity in breast cancer through mitophagy-mediated, ROS-driven p53 pathway activation [493]. Additionally, Sanguinarine has received growing attention for its anticancer effects, including the induction of intrinsic apoptosis and ROS-dependent mitophagy in HCC [494, 495]. Shikonin and its derivatives are known to induce apoptosis, suppress metastasis, and enhance the efficacy of chemotherapy [496–498], Notably, Shikonin triggers mitophagy dysfunction through elevated ROS production, contributing to mitochondrial stress and cancer cell death [498]. These findings demonstrate the therapeutic potential of targeting mitophagy in cancer and support its relevance as both a vulnerability and resistance-modulating mechanism in tumor biology.

## Prospects and future directions

The development of precision therapeutics targeting mitochondrial dysfunction and mitophagy holds significant promise for the treatment of cancer and NDDs. By restoring mitochondrial homeostasis, enhancing or inhibiting mitophagy, and addressing the underlying genetic and molecular defects, these therapies have the potential to greatly improve patient outcomes in these challenging diseases [314, 499, 500]. Lipophilic cationic triphenylphosphonium (TPP⁺)-based conjugates are increasingly used for the selective delivery of compounds to mitochondria, facilitating the targeted modulation of mitochondrial morphology and function. Notably, TPP⁺ conjugates such as 3-carboxy-proxyl nitroxide (Mito-CP) and Metformin (Mito-Met10) have been shown to directly activate ULK1 [501], a key initiator of mitophagy, thereby promoting an anti-tumor mitophagy response. Fisetin conjugates with TPP⁺ group (Mito-fisetin) achieve 100- to 1000-fold accumulation in the mitochondrial compartment and induce anticancer effects by promoting mitochondrial dysfunction and related cytotoxicity [502]. This mitochondria-specific modification highlights the potential of mitochondria-targeted therapeutics in cancer treatment, where mitophagy activation could serve as a strategic intervention.

Moreover, alkyl-TPP modifications can induce reversible, dose- and hydrophobicity-dependent alterations in mitochondrial morphology and function [503]. Emerging selective USP30 inhibitors offer a promising strategy for enhancing the clearance of damaged mitochondria through mitophagy in NDDs, providing a more targeted approach to managing the condition [314, 462–464]. By focusing on selectively modulating mitophagy in affected tissues, this strategy has the potential to revolutionize treatment regimens. Such targeted therapies would not only correct mitochondrial dysfunction but also minimize off-target effects, ensuring that healthy tissues remain unharmed while diseased cells are effectively treated. This shift toward precision medicine could lead to a new era of more personalized and efficient therapeutic options, addressing the underlying pathophysiology of these complex diseases. The promise of tissue-specific mitophagy targeting marks a significant advance in the pursuit of tailored, patient-centered care for both cancer and NDDs. Biomaterials that mimic the extracellular matrix of the brain are widely utilized as carriers for delivering drugs and other biological agents to the central nervous system. The integration of mitophagy modulators with brain-targeted biomaterials holds significant potential to enhance therapeutic efficacy within neural tissues [504, 505]. Recently, mitochondrial luminoptogenetics (mLumiOpto) was designed to be an innovative gene therapy strategy to selectively disrupt the inner mitochondrial membrane potential and induce mitophagy in cancer cells. This approach leverages cancer-targeted exosome-associated adeno-associated virus (AAV) delivery to ensure precise targeting. Preclinical studies have demonstrated that mLumiOpto exhibits significant anti-tumor efficacy, effectively reducing tumor burden and inducing cancer cell death in glioblastoma and TNBC xenograft mouse models [506]. Mitophagy-targeted gene therapy may be a promising therapeutic strategy by targeting the damaged mitochondria of NDDs and cancer.

In conclusion, it’s evident that the aging process in human society has not been a healthy one. Upon reaching a certain age, there is a split in human health; some patients get cancer, and others NDDs, but rarely both. In this review, an overview of the potential underlying biological mechanisms that explain the proven inverse trend between cancer and NDDs has been extensively detailed and considered. While factors like chronic inflammation, ROS, mutations, and cell death are shared in both cancer and NDDs, mitophagy is where the biology for both diseases converges, potentially explaining their inverse trend. It’s necessary to acknowledge how exploring the biology of mitophagy offers new avenues for advancing our understanding and treatment of cancer and NDDs. By leveraging this knowledge, it is possible to consider the influence of mitophagy to guide future therapeutic avenues and mitigate the effects of both conditions.

## Supplementary Information

Supplementary Material 1

## Data Availability

No datasets were generated or analysed during the current study.

## Abbreviations

AD: Alzheimer's disease
ADM: Acinar-to-ductal metaplasia
AIF: Apoptosis-inducing factor
ALS: Amyotrophic lateral sclerosis
AMBRA1: Autophagy and beclin 1 regulator 1
AML: Acute myeloid leukemia
AMPK: AMP-activated protein kinase
Apaf-1: Apoptotic protease activating factor-1
APOE ε4: Apolipoprotein E ε4
APP: Amyloid precursor protein
APP-CTFs: APP C-terminal fragments
ARF1: ADP-ribosylation factor 1
ARIH1: Ariadne RBR E3 ubiquitin protein ligase 1
ATF1: Activating transcription factor 1
ATF6: Activating transcription factor 6
ATG12: Autophagy related 12
ATG5: Autophagy related 5
ATP13A2: ATPase cation transporting 13A2
Aβ: Amyloid-beta
Bak: Bcl-2 antagonist/killer
Bax: Bcl-2-associated X protein
BCAA: Branched-chain amino acid
BCAT2: Branched-chain amino acid transaminase 2
Bcl-1: Beclin-1
Bcl-2: B-cell lymphoma 2
Bcl-xL: B-cell lymphoma-extra large
BCL2L13: Bcl2-like 13
BECN1: Beclin 1
bFGF: Basic fibroblast growth factor
Bid: BH3 interacting-domain death agonist
Bim: Bcl-2 interacting mediator of cell death
BMAL1: Brain and muscle arnt-Like 1
BNIP3: Bcl2-interacting protein 3
BNIP3L, NIX: Bcl2-interacting protein 3-like
bp: Base pairs
C/EBPδ: CCAAT/enhancer-binding protein Delta
C9orf72: The chromosome 9 opening reading frame 72
Ca^2+^: Calcium
CAF: Cancer associated fibroblasts
CALCOCO2: Calcium binding and coiled-coil domain 2
cAMP: The cyclic AMP
CD44: Cluster of differentiation 44
cGAS: The cyclic GMP–AMP synthase
CHMP2B: Charged multivesicular body protein 2B
CHOP: C/EBP homologous protein
CI: Respiratory complex I
CNS: The central nerve system
CP: 3-Carboxy-proxyl nitroxide
CPZ: Cuprizone
CRC: Colorectal carcinoma
CREB: cAMP response element-binding protein
CSCs: Cancer stem cells
CTLA-4: Cytotoxic T-lymphocyte-associated protein 4
CVD: Cardiovascular disease
DAMPs: Damage-associated molecular patterns
DANs: Dopaminergic neurons
DCT-1: DAF-16/FOXO-controlled germline-tumor affecting 1
DCTN1: Dynactin subunit 1
DDR: DNA damage response
DISC1: Disrupted in schizophrenia 1
DJ-1, PARK7: Parkinsonism Associated deglycase
DNM1L: Dynamin-1-like protein
DPD: Deoxypyridinoline
DRP1: Dynamin-related protein 1
EGF: Epidermal growth factor
eIF2α: Eukaryotic translation initiation factor 2 subunit alpha
EMT: Epithelial-mesenchymal transition
EndoG: Endonuclease G
ER: Endoplasmic reticulum
ER-mito: Endoplasmic reticulum–mitochondria contact sites
ETC: The electron transport chain
EVs: Extracellular vehicles
FA: Fatty acid
FAO: Fatty acid oxidation
FBXO7: F-box only protein 7
FC: The frontal cortex area 8
FIG4: FIG4 phosphoinositide 5-phosphatase
FIS1: Mitochondrial fission 1
FKBP8: FKBP prolyl isomerase 8
FoxO: Forkhead box class O
FUNDC1: FUN14 domain-containing 1
FUS: Fused in sarcoma
GBA1: Glucocerebrosidase beta 1
GBM: Glioblastoma
Gp78: Glycoprotein 78
GSH: Glutathione
H₂O₂: Hydrogen peroxide
HCC: Hepatocellular carcinoma
HD: Huntington's disease
HIF-1α: Hypoxia-inducible factor 1α
hiPSCs: Human induced pluripotent stem cells
hMSCs: Human mesenchymal stem cells
HNSCC: Head and neck squamous cell carcinoma
HSPA1L: Heat shock protein family A (Hsp70) member 1-Like
HTT: The huntingtin gene
IL-10: Interleukin-10
IL-1β: Interleukin 1β
IL-6: Interleukin 6
IMM: Inner mitochondrial membrane
IP₃Rs: Inositol 1,4,5-trisphosphate receptors
iPD: Idiopathic Parkinson’s disease
iPSC: Induced pluripotent stem cell
IRE1: Inositol-requiring kinase 1
ITGB4: Integrin β4
kbp: Kilobase pairs
KC mice: Pdx1-Cre mice
KO: Knock out
KRAS: Kirsten rat sarcoma viral oncogene homolog
KRAS*: Mutant KRAS
LC3: Microtubule-associated protein 1 light chain 3
LC3-II: Microtubule-Associated Protein 1A/1B-LC3
LC3B, MAP1LC3B: Microtubule-associated proteins 1A/1B light chain 3B
LIR: LC3-interacting region
LRRK2: Leucine-rich repeat kinase 2
LSCs: Leukemic stem cells
LSL: LoxP-stop-loxP structure
LTCCs: L-type Ca^2^⁺ channels
MAMs: Mitochondria-associated membranes
MAPK: Mitogen-activated protein kinases
Mcl-1: Myeloid cell leukemia 1
MCU: The mitochondrial calcium uniporter
mDA: Midbrain dopaminergic
MDSCs: Myeloid-derived suppressor cells
MERCSs: Mitochondria- endoplasmic reticulum (ER) contact sites
MFN2: Mitofusin 2
mHTT: Mutant huntingtin gene or protein
MIPS: Mitochondrial information processing system
Mito-CP: TPP^+^-conjugated 3-carboxyl proxyl (CP) nitroxide
Mito-Met10: TPP ^+^ -conjugated metformin
MM: Multiple myeloma
MMPs: Matrix metalloproteinases
MOMP: Mitochondrial outer membrane permeabilization
MPP: Mitochondrial processing peptidases
mPTP: Mitochondrial permeability transition pore
MQC: Mitochondrial quality control
MRGs: Mitophagy-related genes
MT: Mitochondrial transfer
mtAP: Mitochondrial antigen presentation
mtDNA: Mitochondrial DNA
MTFR2: Mitochondrial fission regulator 2
mTOR: Mechanistic target of rapamycin
mtROS: Mitochondrial ROS
MUL1: Mitochondrial ubiquitin ligase 1
NADPH: Nicotinamide adenine dinucleotide phosphate
NAM: The mitochondria-associated nucleus structure
NANOG: Nanog Homeobox
NBR1: Neighbor of Brca1
NDDs: Neurodegenerative diseases
NDP52: Nuclear domain 10 protein 52
NF-κB: Nuclear factor kappa B
NFATc: Nuclear factor of activated T-cells, cytoplasmic
NIPSNAP1: Nipsnap homolog 1
NIPSNAP2: Nipsnap homolog 2
NK cells: Natural killer cells
NMJ: The neuromuscular junction
NSCLC: Non-small cell lung cancer
NUMTS: Nuclear mitochondrial DNA
OCT4: Octamer-binding protein 4
OMM: The outer mitochondrial membrane
OPA1: Optic atrophy 1
OPCS: Oligodendrocyte precursor cells
OPTN: Optineurin
OTUD6A: The ovarian tumor-associated protease Deubiquitinase 6A
OXPHOS: Oxidative phosphorylation
PanIN: Pancreatic intraepithelial neoplasia
Parkin: Parkin RBR E3 ubiquitin-protein ligase
PARL: Presenilin-associated rhomboid-like protein
PAS: Pre-autophagosomal structure
PD: Parkinson's disease
PD-1: Programmed cell death protein 1
PD-L1: Programmed cell death ligand 1
PDAC: Pancreatic ductal adenocarcinoma
PDGF: Platelet-derived growth factor
PDGFα: Platelet-derived growth factor alpha
PDGFβ: Platelet-derived growth factor beta
Pdx1: The pancreatic and duodenal homeobox 1
PERK: Protein kinase R-like ER Kinase
PFN1: Profilin-1
PGAM5: Phosphoglycerate mutase family member 5
PGC-1α: Peroxisome proliferator-activated receptor gamma coactivator-1 α
PHB2: Prohibitin 2
PI3K: Phosphoinositide 3-kinase
PINK1: PTEN-induced kinase 1
PKA: Protein kinase A
PKB, AKT: Protein kinase B,
PPP: The pentose phosphate pathway
PRKN: Parkin RBR E3 ubiquitin-protein ligase
Rheb: Ras homolog enriched in brain
RHOT1: Ras homolog family member T1
ROS: Reactive oxygen species
SAD: Sporadic Alzheimer’s disease
scRNA-seq: Single cell RNA sequencing
SIAH1: Seven in absentia homolog 1
Sig1R: The Sigma-1 receptor
Sirt1: Sirtuin 1
SMURF1: SMAD-specific E3 ubiquitin protein ligase
Snapin: SNARE-associated protein
SNc: The substantia nigra pars compacta
SNCA: Synuclein alpha encoding α-syn protein
SOD: Superoxide dismutase
SOD1: Zn/Cu superoxide dismutase 1
SOX2: SRY-box transcription factor 2
Sp1: Specificity protein 1
SQSTM1, p62: Sequestonsome 1
STING: Stimulator of interferon genes
SV: Synaptic vesicle
TAM: Tumor-associated macrophage
tau: Tubulin associated unit
TAX1BP1: Tax1 binding protein 1
TBK1: TANK-binding kinase 1
TCA: The tricarboxylic acid cycle
Tconv: Conventional T cell
TDP-43: TAR DNA binding protein 43 kDa
TFEB: Transcription factor EB
TGF-β1: Transforming growth factor-β1
Th1: T helper 1
Th17: T helper 17
Th2: T helper 2
TILs: Tumor-infiltrating T lymphocytes
TME: Tumor microenvironment
TNBC: Triple-negative breast cancer
TNFα: Tumor necrosis factor-alpha
TNT: Tumor-derived tunneling nanotubes
TNTs: Tunneling nanotubes
TOM20: Translocase of outer mitochondrial membrane 20
TPP⁺: Lipophilic cationic triphenylphosphonium
Treg: Regulatory T cell
TSPO: Translocator protein
TUBA4A: Tubulin alpha 4A
UA: Urolithin A
Ub: Ubiquitin
UBQLN2: Ubiquilin-2
ULK1: Unc-51-like kinase
UPR^mt^: The mitochondrial unfolded protein response
USP30: Ubiquitin-specific protease 30
VAPB: Vesicle-associated membrane protein-associated protein B
VCP/P97: Valosin-containing protein
VDAC: Voltage-dependent anion channels
VDAC1: Voltage-dependent anion-selective channel 1
VEGF: Vascular endothelial growth factor
Ψm: Mitochondrial membrane potential
