# Supplementary material for: Mitophagy’s impacts on cancer and neurodegenerative diseases: implications for future therapies
Source: J Hematol Oncol. 2025 Aug 1;18:78. doi: 10.1186/s13045-025-01727-w (PMC12317492; doi:10.1186/s13045-025-01727-w)
Supplement: Supplementary file 1 — Supplementary Material 1 [file 13045_2025_1727_MOESM1_ESM.docx]

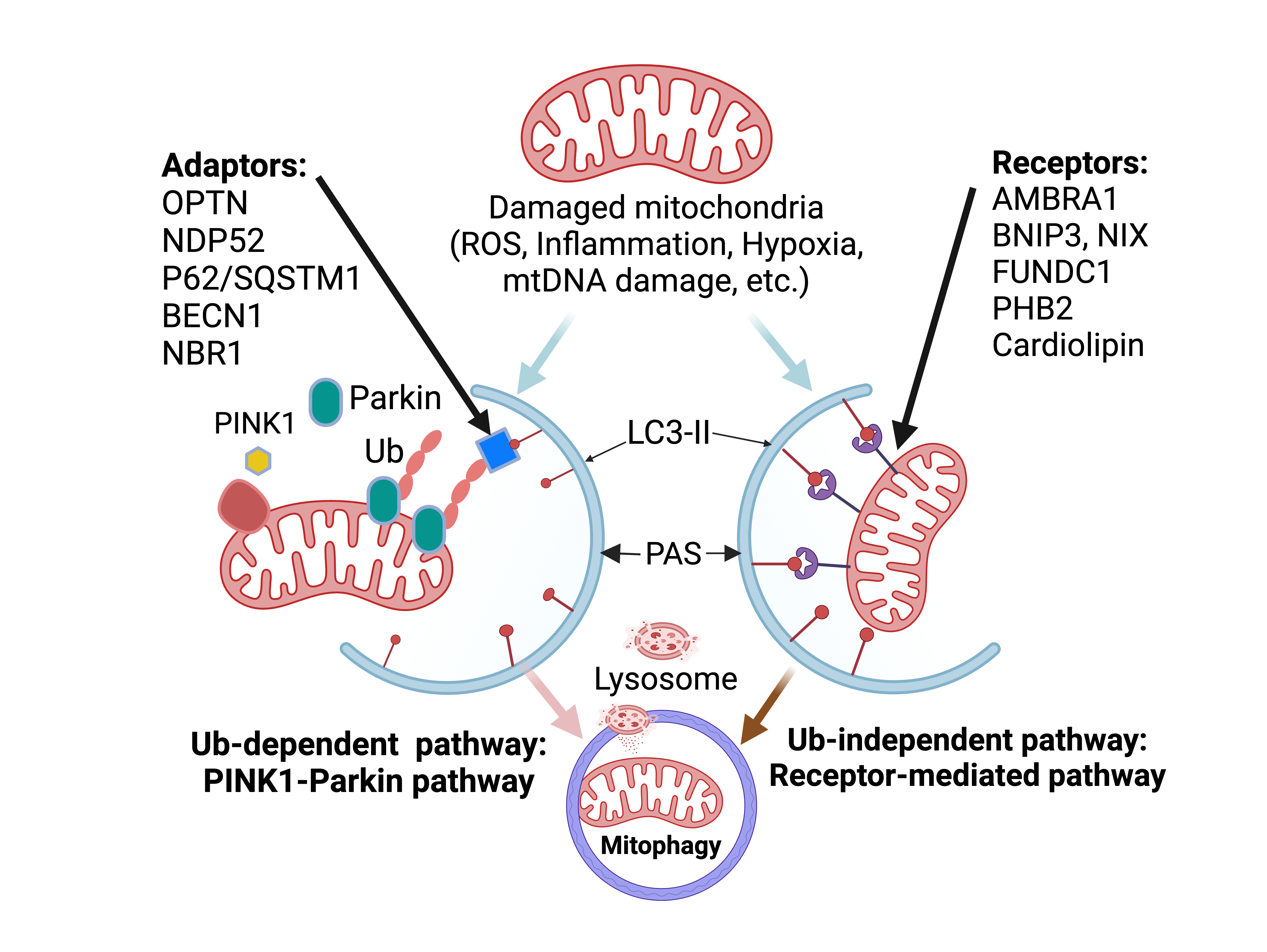


**Figure 1**. **Ubiquitin (Ub)-Dependent and Ub-Independent Mitophagy.**

Damaged mitochondria can result from various factors, including mitochondrial DNA (mtDNA) damage, reactive oxygen species (ROS), hypoxia, inflammation, pathogenic proteins, and both genetic and environmental insults, which are commonly associated with the progression of neurodegenerative diseases (NDDs) and cancer. These damaged mitochondria can be removed through Ub-dependent and Ub-independent mitophagy mechanisms. In the Ub-dependent pathway, mitochondrial outer membrane proteins (e.g., MFN1/2, VDAC1, TOM20) are ubiquitinated by Parkin, which is activated by PINK1. This tagging of proteins leads to the recruitment of Ub-binding mitophagy adaptors (e.g., OPTN, NDP52, P62/SQSTM1, BECN1, NBR1), which then interact with LC3 on autophagosomes, facilitating the engulfment and degradation of the damaged mitochondria. The Ub-independent pathway involves mitophagy receptors (e.g., AMBRA1, BNIP3/NIX, FUNDC1, PHB2, cardiolipin), which are directly activated by mitochondrial damage and bind to LC3, promoting mitochondrial degradation.


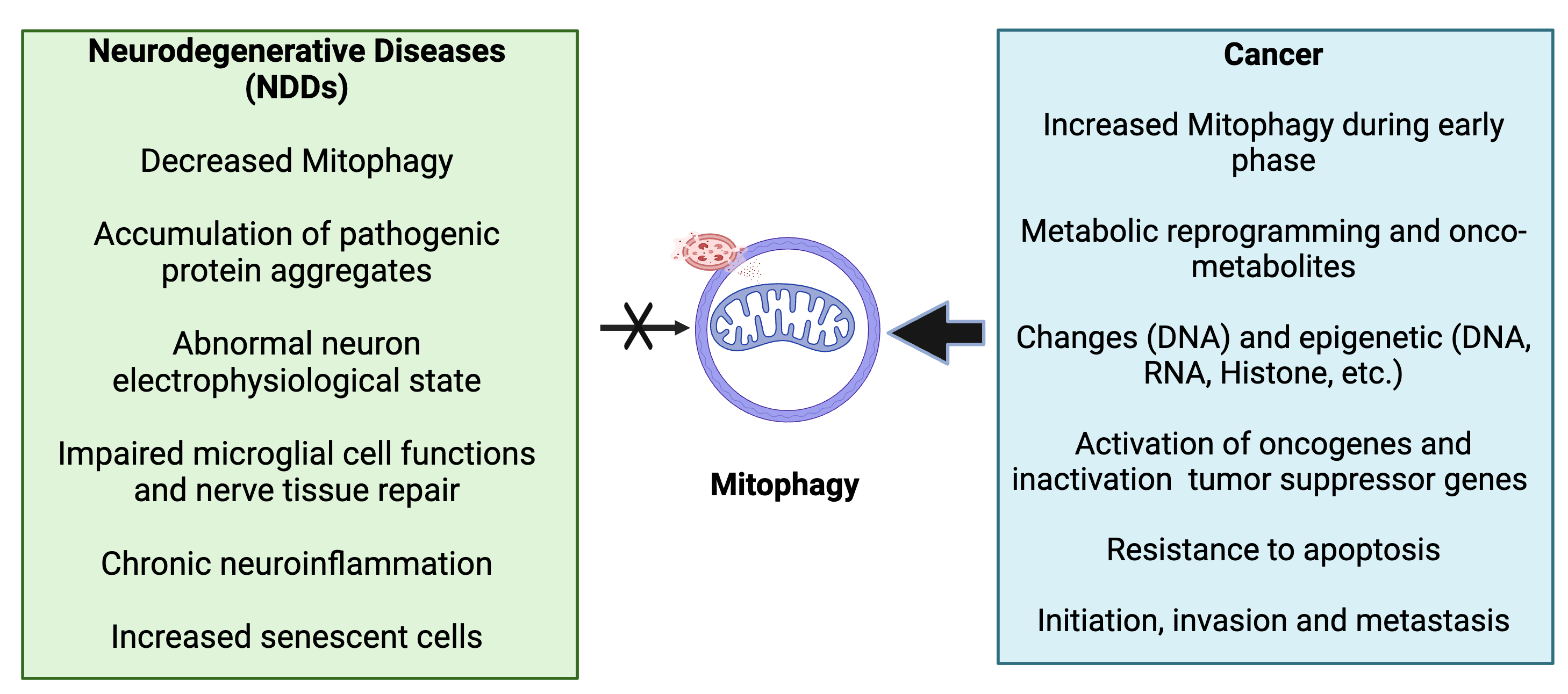


**Figure 2**. **Comparative Analysis of Mitophagy in Neurodegenerative Diseases (NDDs) and Cancers.**

Dysregulated mitophagy is a key contributor to mitochondrial dysfunction in both NDDs and cancer, though with distinct pathological consequences. In NDDs, defective mitophagy leads to the accumulation of dysfunctional mitochondria, exacerbating oxidative stress, synaptic and neuronal network dysfunction, neuroinflammation, and ultimately neuronal injury and death. Conversely, during early tumorigenesis, mitophagy serves as a protective mechanism by clearing damaged mitochondria, thereby preventing ROS production, genomic instability, metabolic and proteomic imbalances, and aberrant signaling factors that drive malignant transformation. However, in established malignancies, mitophagy undergoes context-dependent adaptation, either upregulated or suppressed, to support tumor growth, metastasis, and resistance to therapy.


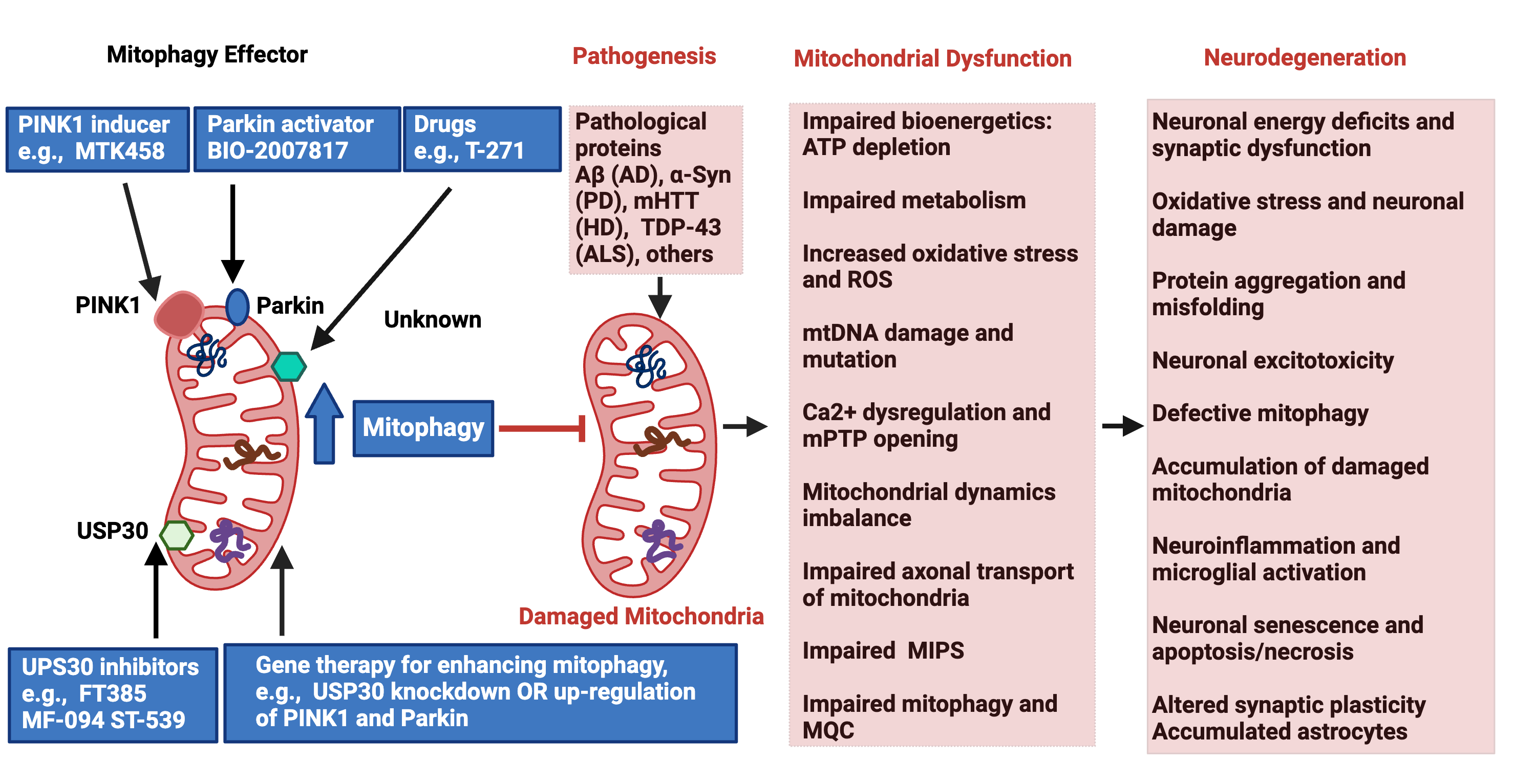


**Figure 3.** **Therapeutic Potential of Targeting Mitophagy in Neurodegenerative Diseases (NDDs)**.

Neurodegenerative diseases (NDDs) are characterized by progressive neuronal damage and loss, often accompanied by chronic neuroinflammation, reactive gliosis, and the accumulation of pathogenic protein aggregates. Mitochondrial dysfunction, particularly impaired mitophagy, plays a central role in these pathological processes by contributing to oxidative stress, bioenergetic failure, and cellular toxicity. Emerging therapeutic strategies aimed at enhancing mitophagy are being actively explored and hold significant promise for mitigating disease progression and improving clinical outcomes in NDDs.


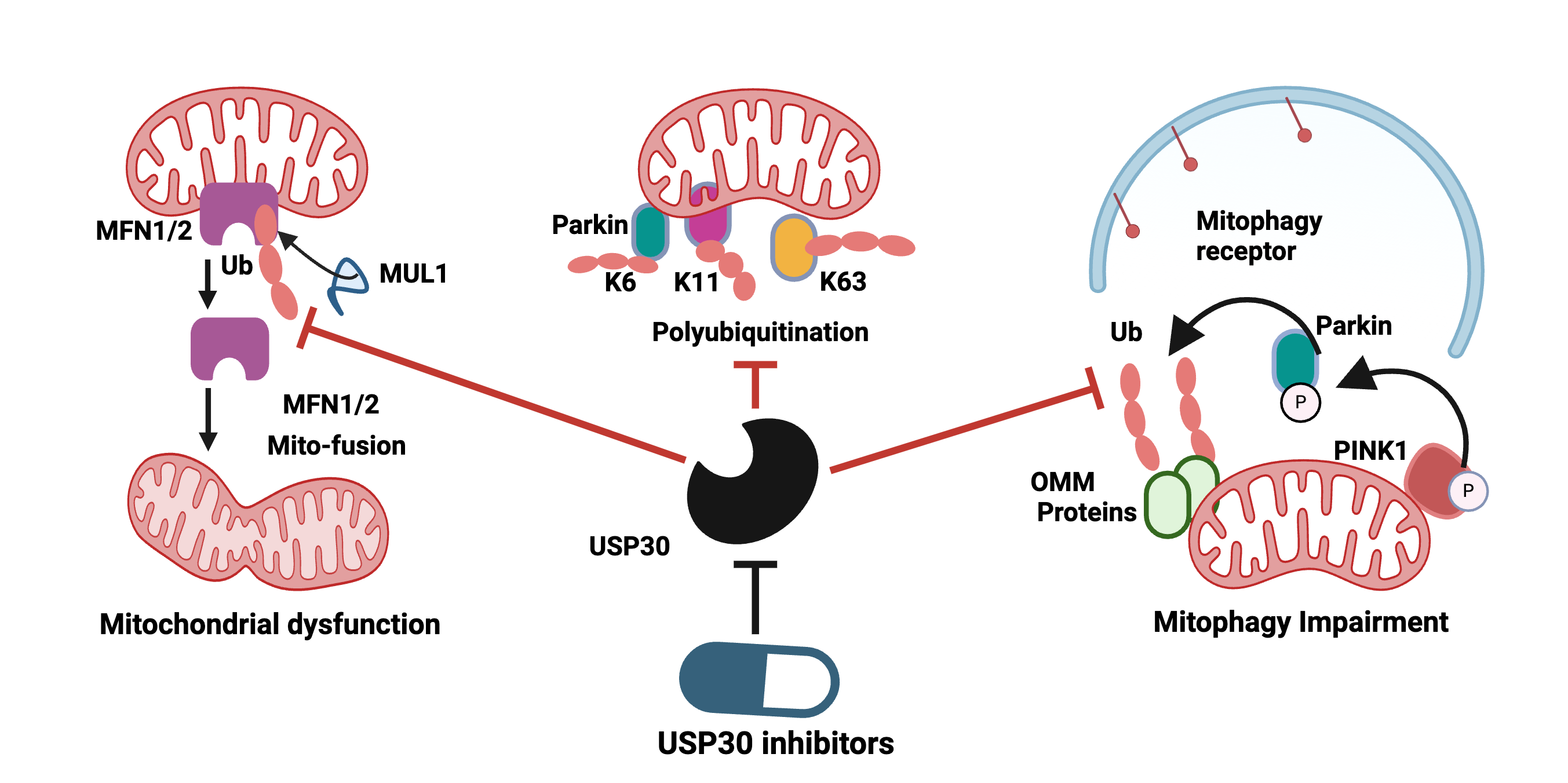


**Figure 4. The Role of Ubiquitin-Specific Protease 30 (USP30) in Mitophagy**.

USP30 regulates mitophagy by modulating mitochondrial dynamics and ubiquitination of outer mitochondrial membrane (OMM) proteins. Specifically, USP30 deubiquitinates Mitofusin 1 and 2 (MFN1/2), increasing their stability and promoting mitochondrial fusion. Additionally, USP30 selectively removes both non-canonical (K6- and K11-linked) and canonical (K63-linked) ubiquitin chains from Parkin and key OMM proteins such as Tom20. This process impairs Parkin recruitment, prevents the activation of mitophagy-related OMM proteins, and ultimately suppresses mitophagy, thereby inhibiting the clearance of damaged mitochondria. Pharmacological inhibition of USP30 has been proposed as a therapeutic strategy to enhance mitophagy, facilitating the removal of dysfunctional mitochondria and restoring mitochondrial function.


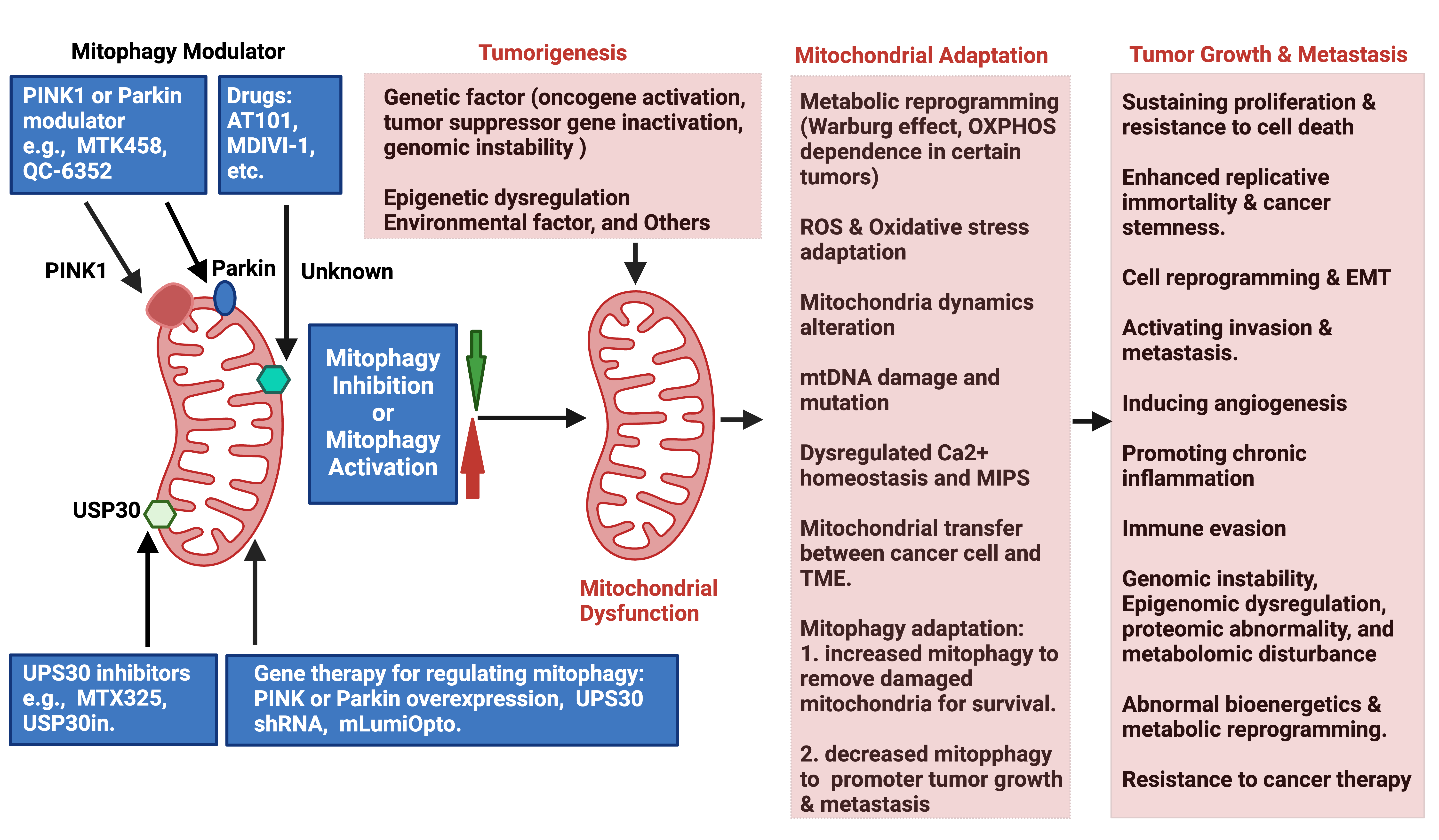


**Figure 5. Therapeutic Potential of Targeting Mitophagy in Cancer**.

Tumorigenesis is a highly complex pathological process driven by genetic and environmental factors, including oncogenic activation, inactivation of tumor suppressor genes, DNA damage, and gene dysregulation. These events frequently lead to mitochondrial dysfunction, resulting in the accumulation of damaged mitochondria and the activation of adaptive mitochondrial responses. A critical component of this adaptation is context-dependent mitophagy, which may be either upregulated or suppressed, ultimately contributing to cancer cell survival, proliferation, metastasis, and resistance to therapy—hallmarks of cancer progression. Given the pivotal role of mitophagy in tumor biology, selective mitophagy-targeting therapeutic strategies are being actively investigated and hold significant promise for improving cancer treatment outcomes.
